# Cryo-electron tomography of the onion cell wall shows bimodally oriented cellulose fibers and reticulated homogalacturonan networks

**DOI:** 10.1016/j.cub.2022.04.024

**Published:** 2022-06-06

**Authors:** William J. Nicolas, Florian Fäßler, Przemysław Dutka, Florian K.M. Schur, Grant Jensen, Elliot Meyerowitz

**Affiliations:** 1 Division of Biology and Biological Engineering, California Institute of Technology, 1200 California Boulevard, Pasadena, CA 91125, USA; 2 Howard Hughes Medical Institute, 4000 Jones Bridge Road, Chevy Chase, MD 20815, USA; 3 Institute of Science and Technology Austria (ISTA), Am Campus 1, 3400 Klosterneuburg, Austria; 4 Division of Chemistry and Chemical Engineering, California Institute of Technology, 1200 California Boulevard, Pasadena, CA 91125, USA; 5 Department of Chemistry and Biochemistry, Brigham Young University, Provo, UT 84602, USA

**Keywords:** onion, cryo-tomography, cell wall, cellulose, pectins

## Abstract

One hallmark of plant cells is their cell wall. They protect cells against the environment and high turgor and mediate morphogenesis through the dynamics of their mechanical and chemical properties. The walls are a complex polysaccharidic structure. Although their biochemical composition is well known, how the different components organize in the volume of the cell wall and interact with each other is not well understood and yet is key to the wall’s mechanical properties. To investigate the ultrastructure of the plant cell wall, we imaged the walls of onion (*Allium cepa*) bulbs in a near-native state via cryo-focused ion beam milling (cryo-FIB milling) and cryo-electron tomography (cryo-ET). This allowed the high-resolution visualization of cellulose fibers *in situ*. We reveal the coexistence of dense fiber fields bathed in a reticulated matrix we termed “meshing,” which is more abundant at the inner surface of the cell wall. The fibers adopted a regular bimodal angular distribution at all depths in the cell wall and bundled according to their orientation, creating layers within the cell wall. Concomitantly, employing homogalacturonan (HG)-specific enzymatic digestion, we observed changes in the meshing, suggesting that it is—at least in part—composed of HG pectins. We propose the following model for the construction of the abaxial epidermal primary cell wall: the cell deposits successive layers of cellulose fibers at −45° and +45° relative to the cell’s long axis and secretes the surrounding HG-rich meshing proximal to the plasma membrane, which then migrates to more distal regions of the cell wall.

## Highlights

• The cellulose fibers cluster according to their orientation
• They adopt a bimodal angular distribution
• They are bathed by a meshing that concentrates at the surface of the cell wall
• The meshing is made of homogalacturonans

## Graphical abstract

**Figure:**
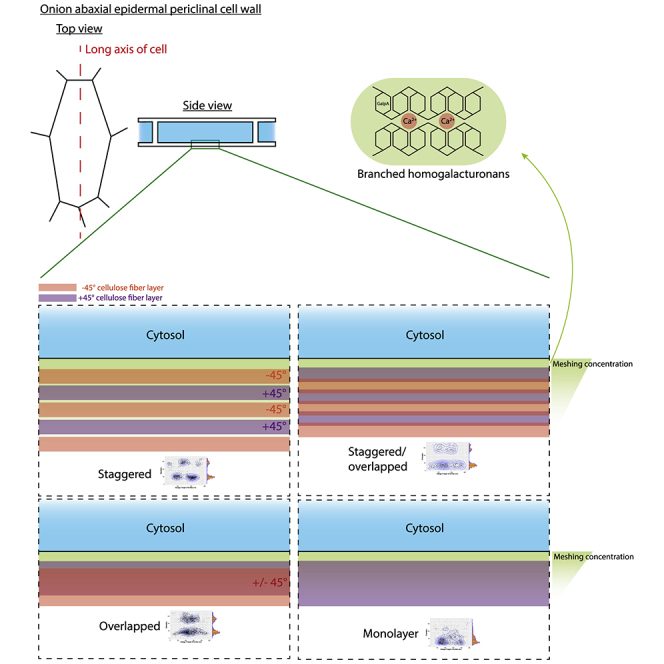

## Teaser

Nicolas et al. used cryo-electron tomography to visualize the primary white onion cell wall at high resolution. The fine organization of the cellulose fibers within the cell wall is characterized, and a mesh-like feature identified as being homogalacturonan pectin is uncovered.

## Introduction

Plants dominate the earth’s biomass^1^ and provide the oxygen necessary for nearly all life on earth through photosynthesis. Photosynthesis allows the fixation of CO_2_ to form simple sugars through the Calvin-Benson cycle, breaking down water molecules and releasing oxygen.^2^ A major fraction of the synthesized simple sugar is used to build up the plant cell wall.^3^ The cell wall is mix of cellulose fibers, pectins, and hemicelluloses, the last two being very chemically diverse.^4^^,^^5^ The complex composite structure of the cell wall is crucial for shaping cells and their function. The unique feature of the cell wall in this context is its ability to resist chemical/enzymatic treatments and mechanical stress while still allowing cells to grow.^5^

The major player in cell shape determination is cellulose. Cellulosic glucan chains assemble to form higher-order fibers with amorphous and crystalline regions.^6^, ^7^, ^8^ The inherent propensity of cellulose fibers to bundle and their modulation, mainly by their interaction with hemicelluloses and pectins, are thought to be very important as they confer additional, higher-order mechanical properties.^9^^,^^10^ The cellulose fibers are secreted into the cell wall by membrane-embedded hexameric cellulose synthase complexes (CSCs), each protomer comprising a trimer of cellulose synthases (CESAs).^11^^,^^12^ In the current model, each CESA secretes a glucan chain, resulting in an elementary fibril secreted by a CSC that is composed of 18 glucan chains. It has been shown that the mature CSCs, upon delivery at the plasma membrane, associate with cortical microtubules via intermediary partners such as cellulose synthase interactive protein 1 (CSI1), which then guide the direction of cellulose synthesis *in muro*.^13^ Although microtubule-guided cellulose synthesis is the most described and well-understood facet of this process, a microtubule-independent pathway has been characterized where CSCs separate from their microtubule track in favor of following an already existing cellulose fiber on the other side of the plasma membrane.^14^ The latter relies on integrating the newly synthesized fibers into an already existing bundle of microfibrils in the cell wall, also hinting toward a mechanism where the motile force of the CSCs is not cytoskeleton-dependent but rather propulsion due to cellulose crystallization.^15^ A cohort of studies showed that the orientations of the cellulose fibers are consequential to the shape of a cell^16^, ^17^, ^18^, ^19^ and the existence of a mechanical feedback loop where the cell is able to sense mechanical cues through its cortical microtubular network and adapt the cellulose fiber patterns in the cell wall.^20^

While the cellulose fibers are thought to be the main load-bearing structures in the cell wall, pectins and hemicelluloses interact with them in ways still not fully understood. Hemicelluloses were hypothesized to bridge cellulose bundles together and form load-bearing hotspots,^21^^,^^22^ although cellulose bundles have been shown to form without the presence of xyloglucan.^23^ Pectins, mainly homogalacturonans (HGs), which comprise up to 60% of the dry weight of the primary cell wall,^4^ are hypothesized to surround all other components and act as a matrix.^8^ Composition, methylation state, and calcium levels have been shown to change the mechanical properties of pectins by altering the level of cross-linking.^24^^,^^25^

Despite our knowledge of the chemical composition of the cell wall and of the diversity of the individual components, structural understanding of their secretion and interaction in the cell wall is underexplored. Cellulose-specific stains have been applied directly to live tissue to observe the cellulose fibers and follow their fate during cell elongation,^18^^,^^26^ but light microscopy does not offer the necessary resolving power to observe the cellulose fibers and their partners at nanometer resolution. White onion (*Allium cepa*) abaxial epidermal cell wall peels have been used in conjunction with high-resolution atomic force microscopy (AFM) and field emission scanning electron microscopy to characterize the organization of the cell wall components at higher resolution.^9^^,^^20^^,^^22^^,^^27^^,^^28^ Despite the knowledge gained, AFM can only access the superficial layers of the cell wall, at best ∼200 nm deep when the surface layers are digested away,^10^ leaving the rest of this polylamellate structure, estimated to be as much as 100 layers, unobserved. Having access to the depth of the cell wall allows a better structural understanding of the cell wall and its relation to cell shape. Here, we used cryo-focused ion beam milling (cryo-FIB milling) followed by cryo-electron tomography (cryo-ET) to observe plunge-frozen *Allium cepa* abaxial periclinal cell walls of onion scale epidermal cells throughout their depth, in near-native conditions.

The high-resolution data we gathered at multiple depths of the cell wall reveal the coexistence of cellulose fibers and a structure coined “meshing,” which our data suggest is made at least in part of HG pectins. The fibers are shown to adopt a bimodular angular distribution creating layers of fibers of alternating angles of ±45° relative to the cell’s long axis.

## Results

### Cryo-ET on epidermal cell wall peel lamellae allows the visualization of the plant cell wall in near-native conditions

**Figure 1:**
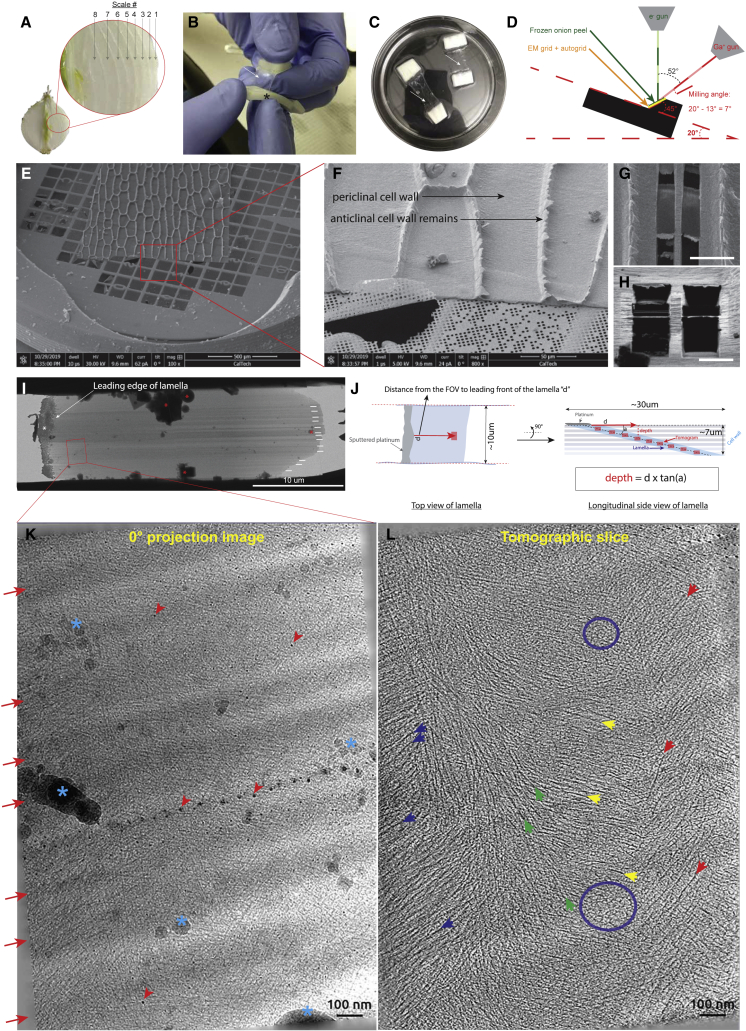
From fresh onion to reconstructed tomograms (A) Half-cut onion showing the concentric scales. The inset shows the classic way the scales are numbered, from outermost to innermost. (B) Process of peeling the abaxial epidermal cell wall. (C) Cell wall peels (clear membranes) attached to the two thicker handles (white) incubating in HEPES. (D) Diagram of the SEM chamber and the position of the onion cell wall peel (green) relative to the FIB and electron beam. (E) SEM overview of a cell wall peel laid on an EM Quantifoil grid. (F) Magnified view of the red box in (E) showing the anticlinal and periclinal cell walls (where the milling was done). (G) SEM overview of two final lamellae milled in periclinal cell wall. (H) FIB view of the two same lamellae shown in (G). (I) TEM overview of a milled lamella. Curtaining is visible (white lines) and contamination is seen on the lamella (red asterisks). (J) Left: diagram of a lamella (top view) showing how the distance *d* from tomogram to the leading edge of the lamella is measured. Right: side view of a lamella illustrating how tomograms distributed along the length of the lamella can sample the different layers of the cell wall. (K) 0° projection image of the red boxed area in (I), 0.40 μm below the surface of the cell wall. Various typical FIB milling artifacts are visible: curtaining (red arrows), platinum projections (red arrowheads), and ice contamination (blue asterisks). (L) Central tomographic slice of the same area shown in (K). Numerous fibers are visible (blue, green, yellow, and red arrows) and small patches of short rod-like, branched densities can be seen between the fibers (blue circle).

**Figure 2:**
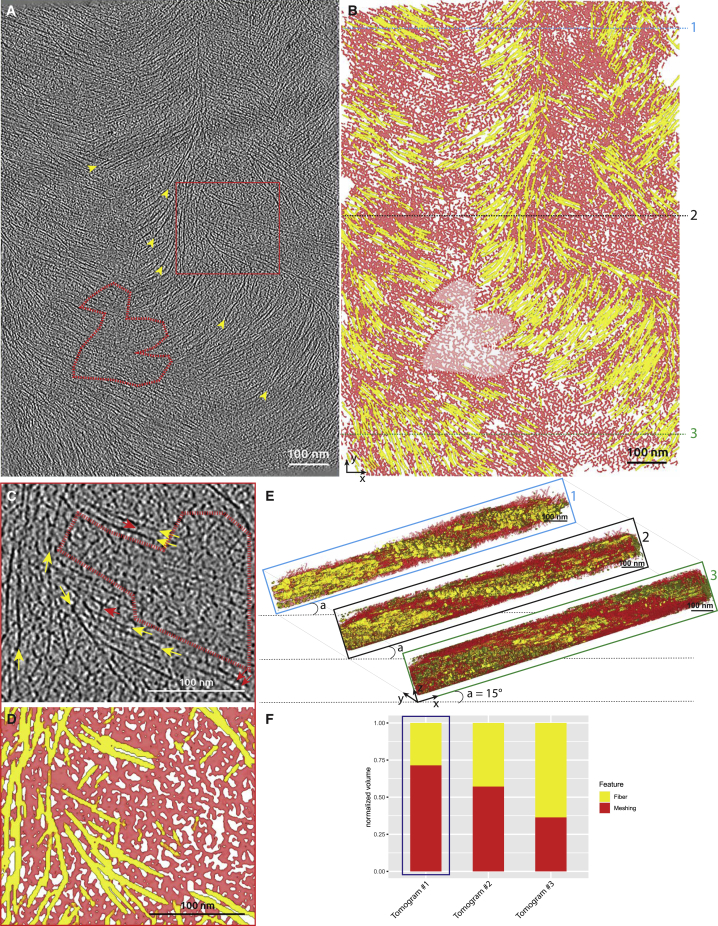
Two features in tomograms of onion cell walls (A) Tomographic slice showing fibers (yellow arrowheads) and the filling material in between the fibers (red dashed region), the meshing. This tomogram originates from scale #6, 0.64 μm below the surface of the cell wall. (B) CNN segmentations of the fibers (yellow) and the meshing (red). (C) Magnified view from the red boxed region (A) showing fibers (yellow arrows), bridges between the fibers (red arrows), and patches of meshing (red dashed region). (D) CNN segmentation of the magnified region. (E) Composite image showing transverse views at different Y-levels of the segmented volume shown in (B). Tilting is the correction for the inclination of the lamella relative to that of the wall. Alternations of fibers (yellow) and meshing (red) are observed. (F) Relative occupancy of fibers versus meshing in three tomographic volumes equivalent to the one shown in this figure (blue squared column is the tomogram shown in this figure). See also Figures S1 and S2 and Video S1.

White onion cell wall peels from the concentric scales, numbered from 1 (outermost and oldest scale) inward to number 8 (innermost youngest scale), were generated as described previously (Figures 1A–1C).^29^^,^^30^ Cryo-FIB milling was performed (Figure 1D) on flash-frozen periclinal cell walls (Figures 1E and F) to produce lamellae ∼200 nm in thickness, allowing access to the deeper layers (Figures 1G and 1H). As the angle of milling was well defined, it was possible to measure the depth of the tomograms in the cell wall (Figures 1I and 1J). Keeping in mind the known artifacts visible on the lamellae, such as curtaining, surface ice contamination, and surface platinum streaks (Figure 1K, red arrows, blue asterisks, and red arrowheads, respectively), tomographic data acquired in this way allowed visualization of the organization of the different elements in the cell wall at high resolution, in near-native conditions. Fields of fibers organized in arrays (Figure 1L, colored arrows) were observed, as well as small, intercalated patches of thin, reticulated densities we term “meshing” (Figure 1L, blue circles, and Figure 2). At the same time, we found the meshing to intercalate between bundles of cellulose fibers (Figures 2A–2D, yellow arrows pointing to cellulose fibers, red arrows and dashed line pointing to the meshing; Video S1). Because manual segmentation of these two features was impractical, two convolutional neural networks (CNNs) were trained to recognize these two features using EMAN2 software.^31^ The fiber detection CNN, being very specific, yielded precise maps of the fibers (Figures S1A–S1C). However, the meshing detection neural network also detected the fiber densities in the tomogram. To circumvent this issue, subtraction of the CNN fiber map from the meshing-CNN map was performed, which assumes that all densities that are not fibers are associated to the novel meshing (Figure S2). The meshing is seen accumulating in patches between the fibers and connecting the fibers together. Successive X-Z cross-sections of the segmentations corrected for lamella tilt allow qualitative assessment of the distribution of these two features within the volume (Figure 2E). In tomograms with a similar layout as in Figure 2A, the volume occupancy of the meshing versus that of the fibers ranged from 30% to 75% (Figure 2F).

Video S1. Cryo-FIB milling and cryo-ET of onion cell wall peels, related to Figure 2 Cryo-FIB milling allows to generate lamellae in the epidermal onion cell wall peels. Cryo-ET on these lamellae allowed the visualization in 3-dimensions of the cellulose fibers (yellow) and the meshing (red).

### Fibers travel straight and horizontally in the cell wall and adopt a bimodal angular distribution

To produce a vector representation of the fibers suitable for geometrical analysis, a template-matching strategy using the Amira TraceX add-on^32^ was used on the fiber-CNN maps (Figures S1C–S1F).

**Figure 3:**
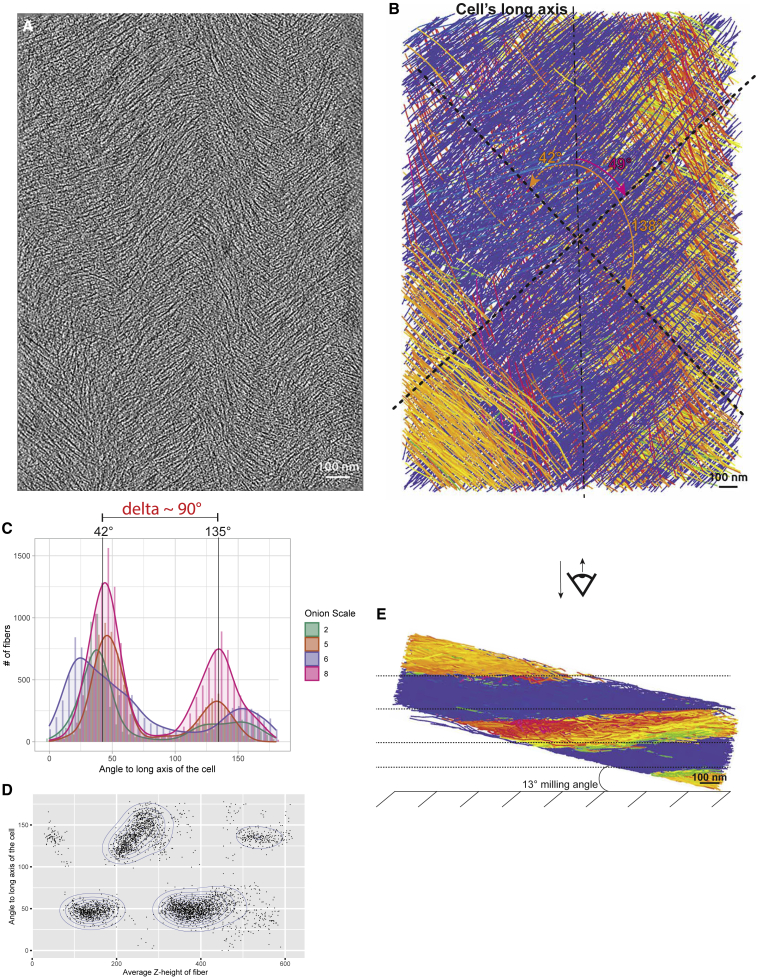
Cellulose fibers organize in a bimodal angular pattern (A) Tomographic slice of the cell wall from scale #8, 3.36 μm below the surface. (B) Automated segmentation of the volume shown in (A). Color coding is according to the clockwise angle of the fiber relative to the cell’s long axis (∼vertical dashed line). The two dashed crossed lines indicate the two main angular modes in this volume: 49° and 138°. The latter is equivalent to a 42° counterclockwise angle. (C) Distribution plot of the angle of the fibers relative to the cell’s long axis by scale number. The 42° and 135° angles correspond to the global modes, aggregating all fibers from all scales. The difference between these two modes is ∼90°. (D) Scatterplot of angles of fibers versus their average height in the tomographic volume shown in (A) and (B). (E) Bottom cross-sectional view of the segmented volume shown in (B). See also Figures S3–S5, Table S1, and Video S2.

The following results were extracted from a total dataset of 31 tomograms acquired across the onion scales #2, 5, 6, and 8 (Figure 1A; Table S1 for a precise description of the data and samples). The density distribution of the orientation of the fibers was analyzed for each tomogram. Twenty-six out of the 31 tomograms considered showed a bimodal distribution (Figures 3A and 3B; Video S2), the five others exhibited a unimodal distribution (Figure S3D). All the tomograms displaying a bimodal distribution had very similar angles to the long cell axis, averaging 42° ± 8° (n = 31 tomograms) and 135° ± 10° (n = 26 tomograms), showing a difference between the two modes of ∼90°. Since all angles were calculated clockwise, the 135° relative to the cell’s long axis is equivalent to a 45° angle counterclockwise (Figures 3B and 3C). This is reminiscent of previous AFM observations by Zhang et al.^33^ When organized according to the scale number where the tomogram was acquired, the density distributions show very similar modes (Figure 3C), suggesting that this bimodal distribution of the orientation of the fibers is consistent throughout all developmental stages studied. Fibers with the same angle cluster together according to their Z-height within the tomographic volume (Figure 3D), creating horizontal layers of cellulose fibers alternating between 45° clockwise/counterclockwise (Figure 3E).

Video S2. The bimodal angular pattern of the cellulose fiber orientations in the onion cell wall peels, related to Figure 3 The cellulose fibers in the tomograms cluster by their orientation relative to the cell’s long axis, creating horizontal layers with following a bimodal angular pattern.

Three more distribution patterns were observed in addition to a perfectly staggered pattern (Figures 3D and S3A): overlapped (12 out of 31 tomograms), with fibers of both modal angles mixed at all heights of the tomographic volume (Figure S3B); staggered-overlapped (nine out of 31 tomograms), similar to the staggered pattern but with overlapping (Figure S3C); and unimodal (five out of 31 tomograms), with only one modal angle (Figure S3D).

The effect of the depth in the cell wall and the aspect ratio of the cell on the angular distribution was investigated on a per-scale basis. The bimodal angular pattern is found throughout all scales studied (#2, 5, 6, and 8) and at all depths and cell aspect ratios where data were acquired (Figures S4A and S4B). The aspect ratios of the milled cells fell into the ranges measured from the light microscopy montages (Figures S4C and S4D, colored vertical lines). The average aspect ratios for scales 2, 5, and 8 and their standard deviation overlap strongly (4.3 ± 1.9, 3.8 ± 1.5, and 4.1 ± 1.7, respectively), suggesting that there is little to no change in the cell’s aspect ratio as the scale is pushed outward during growth of the onion (Figures S4C and S4D).

The straightness and the horizontality (relative to the horizontal plane of the cell wall) of each fiber were also analyzed by computing the average radius of curvature and average slope of each fiber, respectively (see STAR Methods for details on the computation of these parameters). The average radius of curvature measured throughout all the tomograms is 225 ± 90 nm, which suggests that the fibers are overall straight (Figures S5A and S5B). The average slope measured throughout all the tomograms is 0.02 ± 0.4 and is centered around 0 (Figure S5C), indicating that the fibers describe horizontal trajectories within the volume of the wall, which is clearly observable when looking at cross-sections in the segmentations (Figures S5D and S5E). Despite not being detected by our segmentation method, occasional kinked fibers were spotted (Figures S5F–S5I).

In summary, these results show that the fibers organize in layers that harbor orientations describing a bimodal angular pattern, at roughly ±45° relative to the cell’s long axis, are relatively straight, and travel horizontally relative to the cell wall’s horizontal plane.

### The meshing accumulates at the surface of the cell wall

**Figure 4:**
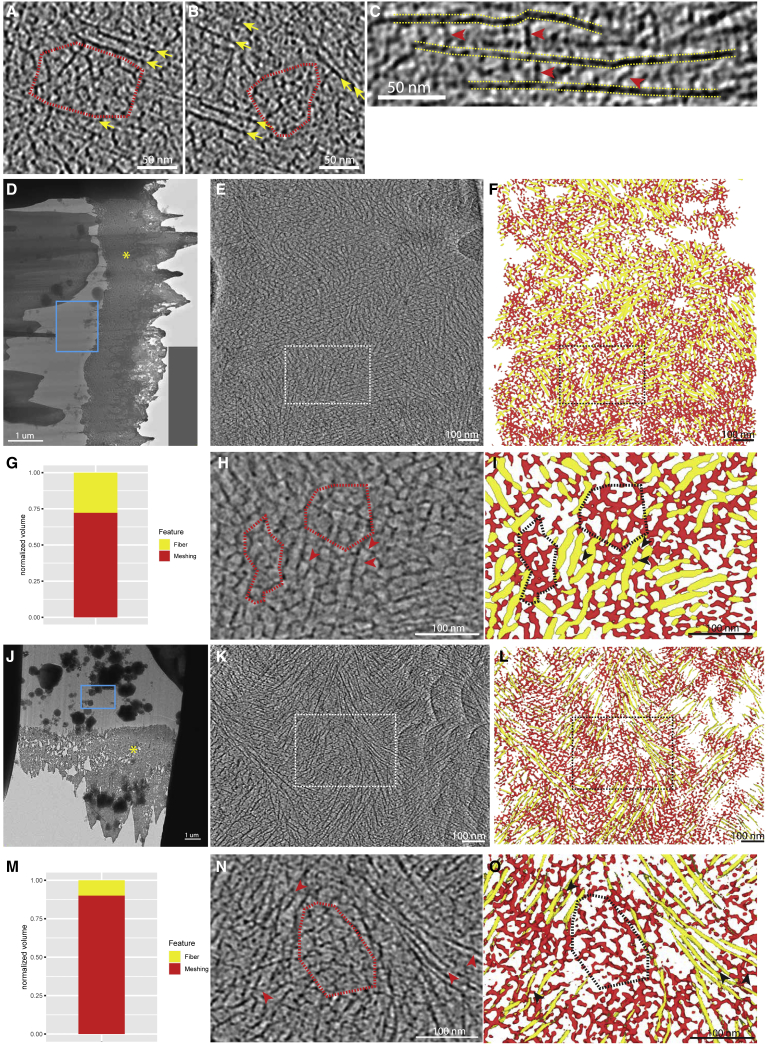
The meshing is seen in patches and also bridging the fibers together (A and B) Examples of small patches of meshing (circled in red) surrounded by fibers (yellow arrows). The meshing is characterized by small, branched segments with no particular orientation, creating a reticulated network. (C) Examples of meshing segments (red arrows) bridging fibers together (yellow dashed lines). (D and J) Overviews of lamellae. Yellow asterisk points to the platinum layer. (E and K) Tomographic slices of tomograms acquired near the top of the cell wall (blue rectangle in D and J, respectively) at 0.15 and 0.55 μm below the surface, respectively. This tomogram is enriched in meshing as many reticulations can be seen. (F and L) Associated segmentation of the tomographic slice shown in (E) and (K), respectively. Meshing is in red and fibers in yellow. (G and M) Relative quantity of meshing versus fibers in the tomogram shown in (E) and (K), respectively. (H and N) Magnified views of the white rectangles shown in (E) and (K), respectively. Examples of patches of meshing are shown (red dashed circles) and events of fiber bridging are highlighted (red arrowheads). (I and O) Corresponding segmentation of the magnified view (H) and (N), respectively. See also Video S3.

**Figure 5:**
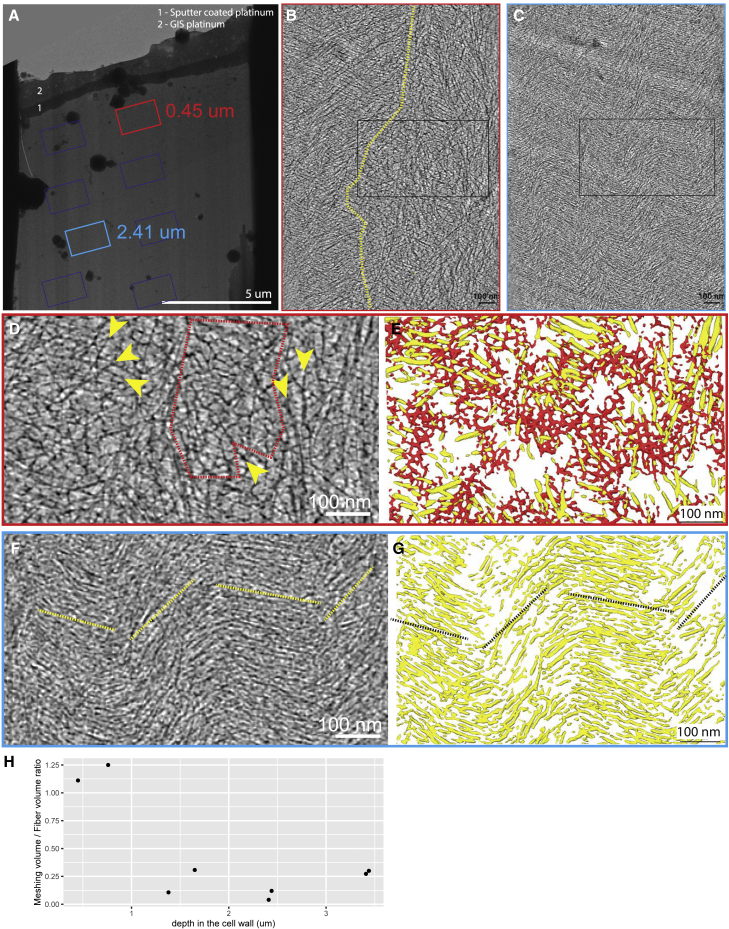
The meshing is concentrated at the top of the cell wall (A) Overview of lamella milled in a non-treated cell wall peel from scale #2. Rectangles show where tilt series were acquired. (B) Tomographic slice acquired near the top of the cell wall (red rectangle in A, at 0.45 μm from the surface). The dashed yellow line indicates the visual limit between an area with a loose network of fibers with a substantial amount of meshing intercalated between the fibers (right of the line) and an area where the fibers seem more bundled together and less meshing is visible (left of the yellow line). (C) Tomographic slice acquired further down the cell wall (blue rectangle in A, at 2.41 μm from the surface). It shows a denser network of fibers with virtually no visible meshing. (D) Magnified view from the black rectangle in (B). Extensive patches of meshing intercalated with fibers can be observed (red dashed region and yellow arrowheads, respectively). (E) Associated segmentation of the magnified view (D). (F) Magnified view from the black rectangle in (C). Tightly packed fibers with very constant orientations are seen. Yellow dashed lines show the general orientation of the layers visible in this tomographic slice. (G) Associated segmentation of the magnified view (F). (H) Meshing versus fiber volume ratio calculated from the CNN segmentations in the eight tomograms extracted from this lamella. The ratios are plotted against the tomogram depth in the cell wall. See also Video S3.

We also characterized the meshing, which takes the form of thin and short fibrous densities that either reticulate, forming a web-like network (Figures 4A and 4B), or bridge cellulose fibers together (Figure 4C). Tomograms acquired proximal to the platinum layer and thus close to the previous wall interface with the plasma membrane (Figures 4D and 4J) and show extended areas of reticulated meshing (Figures 4E, 4F, 4H, 4I, 4K, 4L, 4N, and 4O, red and black dashed delineations) accompanied by a reduction in the concentration of fibers. In regions of enriched meshing, the relative volume of the wall region manifesting meshing can be above 50% (Figures 4G and 4M). Having lamellae milled at an angle allowed probing of the structure of the cell wall, not only proximal to the cell surface but also more distally in the cell wall (Figure 5A). We were therefore able to follow the distribution of the meshing within the depth of the cell wall. Proximal to the cell wall inner surface, transition areas could be observed even within a single tomographic volume. A sub-region of the tomogram was depleted in meshing (Figure 5B, left of the yellow dashed line; Video S3) and contained ordered bundles of fibers, while the other sub-region was enriched in meshing (Figures 5B, 5D, and 5E; Video S3) and exhibited more disordered arrays of fibers. In contrast, tomograms acquired deeper in the cell wall (farther from the plasma membrane) had reduced amounts of meshing and displayed fibers with an increased degree of bundling and order (Figures 5C, 5F, and 5G; Video S3). Quantitative analysis of the segmented meshing volume to segmented fiber volume ratio shows a rapid drop in the amount of meshing at ∼1 μm deep in the cell wall (∼1.25 to ∼0.25 ratio) and then remains stable (Figure 5H).

Video S3. The meshing is found in increased amounts proximal to the leading edge of the lamella, i.e., at the surface of the cell wall, related to Figures 4 and 5 Tomograms taken close to the top of the cell wall are enriched in meshing in comparison to tomograms taken deeper in the cell wall where the cellulose fibers appear to be more ordered with less meshing present.

Taken together, these results suggest the meshing is secreted out of the cell, accumulates at the cell wall-PM interface, and reticulates between the fibers of the first layers of the cell wall.

### Enzymatic digestion of the HGs alters the morphology and abundance of the meshing

We sought to identify the chemical nature of the meshing. The onion outer-epidermal cell wall is composed of 10% xyloglucans (the main hemicellulose) and up to 50% HGs (the main pectin).^34^ In the most recent models of the interactions between the different components of the primary cell wall, pectins are thought to surround and tether the cellulose fibers in a calcium-dependent manner.^8^^,^^10^^,^^24^ We thus hypothesized that the meshing was at high odds of being predominantly composed of HG. The cell wall peels were treated with either BAPTA, a calcium chelator, or *Aspergillus* pectate lyase (PL), an enzyme that digests de-methylesterified HGs, and then processed by cryo-FIB milling and cryo-ET to see whether the previously observed meshing would be morphologically altered. The efficiency of the treatments was verified by staining treated and non-treated peels with chitosan oligosaccharide Alexa 488 (COS488), an HG-specific fluorescent probe (Figures S6A and S6B). The decrease in fluorescence (most apparent in the PL-treated material) suggests that these treatments reduce the pectin content in the peels (Figures S6C–S6F). While applying the onion peels to the EM grids, we noticed that the PL-treated ones seemed to exhibit greatly reduced “stiffness” or, to be consistent with previous rheological studies, increased “loosening” was observed after PL treatment.^9^^,^^10^ Cryo-SEM images showed a clear difference in the aspect of these peels (Figure S7). Indeed, they revealed the topological features of the underlying grid bars and even Quantifoil holes of the carbon (Figures S7D and S7E), and also showed a qualitative reduction in the prominence of the bases of the torn-off anticlinal cell walls (Figure S7D). Being able to visualize what is underneath the peel by cryo-SEM suggests that the specific digestion of demethylated pectins from the cell wall affects cell wall thickness or resistance to bending (Figure S7).

**Figure 6:**
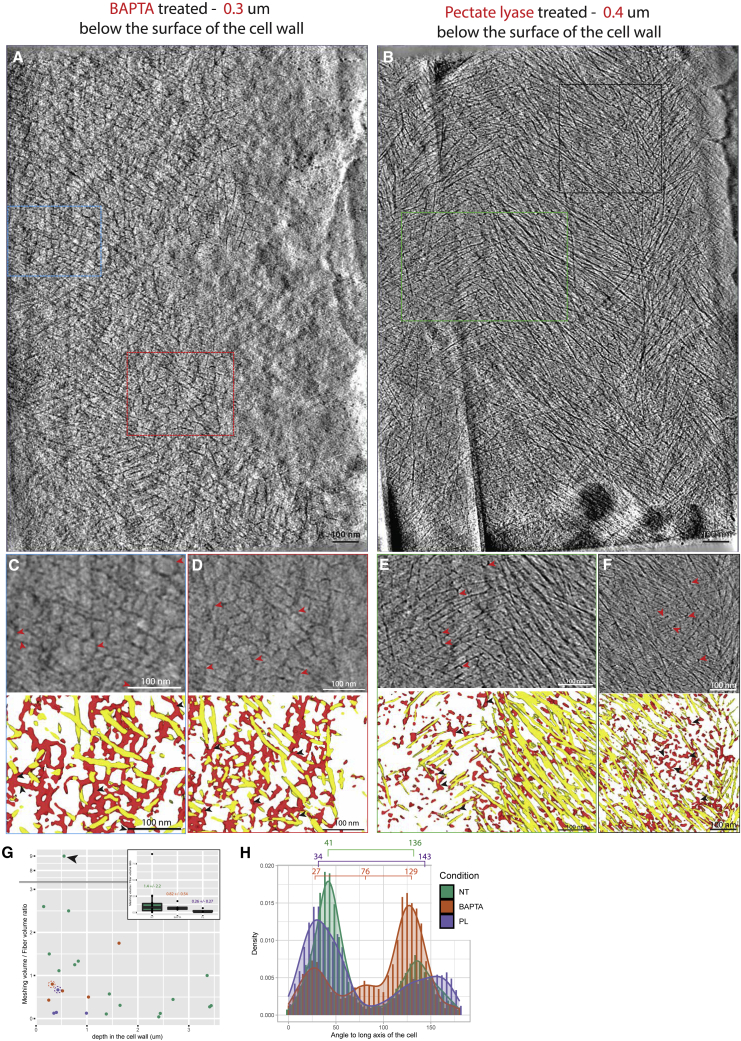
The morphology of the meshing is affected by pectate lyase but not by BAPTA (A) Tomographic slice 0.3 μm under the surface of a BAPTA-treated cell wall. Meshing patches can be seen among the fibers. (B) Tomographic slice 0.4 μm below the surface of a PL-treated cell wall. Meshing remnants can be seen around the fibers. (C and D) Magnified views of areas of the tomogram (blue and red rectangles in tomogram, A, respectively) displaying meshing patches (red arrows) with the associated segmentations. (E and F) Magnified views of areas of the tomogram (green and black rectangles in tomogram, B, respectively) displaying small remnant densities in between the fibers (red arrowheads) with the associated segmentations. (G) Meshing versus fiber volume ratio calculated from the CNN segmentations calculated from 16, 5, and 4 tomograms from non-treated, BAPTA-, and PL-treated peels. Black arrow points to the unusually high meshing/fiber ratio. The inset boxplot shows the mean meshing/fiber ratio for each condition. The orange and purple dashed circles indicate the data points linked to tomograms shown in (A) and (B), respectively. (H) Distribution plot of the angle of the fibers relative to the cell’s long axis by condition. The brackets show the modal values for each of these conditions. See also Figures S6 and S7 and Table S1.

BAPTA-treated cell wall peels show visible meshing, as in untreated walls in increased concentration proximal to the leading edge of the lamella (Figures 6A, 6C, and 6D, red arrowheads). PL-treated cell wall peels show no meshing at all, or remnant densities between the fibers and in small patches that we interpret as incompletely digested meshing or the portion of the meshing insensitive to the specific activity of pectate lyase (Figures 6B, 6E, and 6F). Quantification of the meshing volume versus the fiber volume, as a function of depth of the tomogram in the wall in the non-treated condition, clearly shows a gradual decrease (Figure 6G, average ratio of 0.90 ± 0.82). The unusually elevated ratio (∼9-fold more meshing; Figure 6G, black arrowhead) represented a region of the cell wall ∼500 nm below the surface (tomogram shown in Figures 4J–4O) and was excluded from the computation of the average. The ratios found at the surface of the cell wall in the BAPTA-treated peels show a steady amount of meshing, overall lower than in the same non-treated regions of the cell wall (average ratio of 0.82 ± 0.54). In the PL-treated peels, the ratios were much lower (0.26 ± 0.27). This suggests that PL treatment reduces the amount of meshing and alters its morphology, indeed in some cases making it practically disappear. Angular distribution of fibers was also assessed in the BAPTA-/PL-treated cell wall peels and the bimodal angular distribution pattern was conserved (Figure 6H).

**Figure 7:**
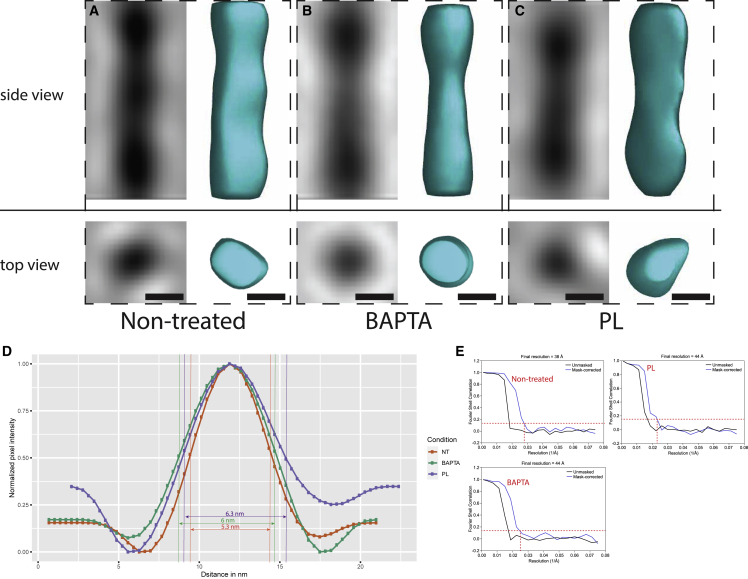
Fiber averages in non-treated and treated conditions (A–C) Side views (top) and top views (bottom) of the fiber averages for the non-treated, BAPTA, and PL condition, respectively. Scale bars, 5 nm. (D) Sideview profiles of the averages shown in (A)–(C) and the FWHM measurements. (E) Fourier shell correlation (FSC) curves (unmasked in black and mask-corrected in blue). Red horizontal line is the 0.143 FSC gold standard value, and the red vertical line indicates the frequency corresponding to this FSC value when mask corrected.

To assess whether the treatments had an impact on the cellulose fiber diameter, averages were generated for each condition (Figures 7A–7C) and their cross-sectional diameters were compared by calculating the full-width at half-maximum (FWHM) on the full-length average density profiles. We were not able to measure a significant difference among the three averages generated (5.3, 6.0, and 6.3 nm cross-sectional diameters for the non-treated, BAPTA, and PL conditions, respectively) (Figure 7D), suggesting the treatments did not alter the diameter of the cellulose fibers bundles.

### Purified pectins reproduce the morphology of wall meshing

To test our hypothesis that this meshing network seen around the fibers in the tomograms, and altered in the presence of PL, is made of HGs, we imaged purified pectins in an aqueous solution. Citrus pectins with an 89% content in galacturonic acid (HG) and a degree of methylation of 38% were viewed using cryo-ET. As a negative control, solvent only (DI water) grids were also prepared. The latter showed no features (Figure S6G), while the HG solution showed reticulated networks reminiscent of the meshing seen in the native cell walls (Figures S6H and S6I, red arrowheads).

## Discussion

### Implications of the bimodal angular distribution

The bimodal angular layering we observed (Video S2) confirms the crossed-polylamellate organization of the onion primary cell wall.^22^^,^^27^ Previous AFM data have observed a similar pattern at the inner surface of the cell walls.^29^^,^^33^ Additionally, previous attempts at observing the fibers of the cell wall in a native state by using cryosectioning were not of sufficient quality (mainly because of imaging equipment) to perform the analysis presented here.^35^, ^36^, ^37^ Our observations, despite the approximate ±10° variation in both modes, show that the ±45° bimodal angular distribution is found ubiquitously at all the scales studied (Figure 3C), at all depths of the cell wall, and at all aspect ratios of the cells observed (Figures S4A and S4B), unlike observations made in previous studies in *Allium cepa* and Arabidopsis.^18^^,^^29^ It cannot be excluded that this discrepancy is due to the history and origin of the onions used, the fundamental difference between onion cells and Arabidopsis root epidermal cells from the elongation zone, and the fact that cell expansion in epidermal onion cells cannot be solely reduced to the 2D aspect ratio of the cell.^29^^,^^38^

### Layering patterns

We observed four different stacking patterns of the cellulose fiber layers (Figure S3), suggesting that multiple factors can influence the trajectories of the cellulose fibers. In the light of this, we suggest that microtubule guidance can initiate a new layer with a different orientation from the previous one, which can then be maintained and reinforced by the microtubule-independent cellulose guidance acting as a positive feedback mechanism.^14^ Creating staggered alternating layers of cellulose fiber bundles ±90° from each other would require very drastic switches between these microtubule-dependent and -independent cellulose synthesis modes. This can either be done by a complete synchronous turn-over of the CSCs, which has been measured to require 8 min,^39^ and a cessation of cellulose synthase activity through phosphorylation,^40^^,^^41^ or a sudden upregulation of CCs and CSI1 translation or delivery to the plasma membrane to redirect CSCs by reattaching them to microtubules so as to create a layer at a new orientation. It remains to be determined how and why the two modes at roughly ±45° are conserved throughout the cell wall. Overlapped events where a mix of orientations are observed at a given height may indicate areas of the cell wall where this sudden directional switch did not occur or failed, hence the gradual transition. “Monolayered” instances (5/31 tomograms; Figure S3D) highlight areas where the cellulose layers are too thick to be entirely captured in one tomographic volume.

### The mechanical relevance of a long-range straight fiber structure

Our results indicate that the cellulose fibers are generally straight, though allowing some bend (Figures S5A and S5B). Our segmentation method, using conjunctly CNNs and template matching, turns out to be very efficient for relatively straight fibers. However, we do not believe that our method of quantifying the radii of curvature would allow efficient recognition of local kinking events that have been identified before.^14^^,^^22^ The search cone used by Amira to trace the fibers on the CNN-segmented fiber maps had an angle of 37°, thus limiting its search to tracing within that range, excluding any fibers with kinks >37°.

Despite this limitation, kinked fibers were seen on rare occasions by visual inspection (Figures S5F–S5I), reminiscent of previous AFM observations.^22^

We were able to assess how the fibers run horizontally relative to the plane of the cell wall (Figures S5C–S5E). This fits well into recent molecular dynamics studies where cellulose protofilaments exiting the CSCs are projected to be in a transient disordered state that later spontaneously polymerizes orthogonally to the plasma membrane. These fibers are then horizontally aligned through the pressure exerted by the existing cell wall and the disorganized clusters proximal to the plasma membrane acting as a flexible hinge.^42^

### The meshing distribution and the mechanical properties of the cell wall

Tomograms of the near-native cell wall show an interconnected network of meshing and cellulose fibers, confirming the single network model of the primary cell wall^43^^,^^44^ (Figure 4). Consistent with previous AFM work that identified a layer of “meshwork” interfacing between the plasma membrane and the surface of the cell wall,^27^ we observed this meshing concentrated at the surface of the cell wall (Figures 5D–5O), but not only here, as it seems to serve as a matrix between the fibers even in more distal layers of the cell wall. A recent molecular dynamics model proposes that cellulose and pectin layers alternate with each other.^45^ This resembles our observations, with the exception that in the hypothetical model, single-pass cellulose layers alternate with layers of pectic matrix, whereas we observed much thicker layers (Figures 2E and 3E; Video S2). In our experiments, the meshing is detectable up to ∼1 μm deep in the cell wall, before the meshing to fiber ratio suddenly drops below 0.5 (Figures 5H and 6G), appearing as smaller patches and inter-fibrillar bridges (Figures 2 and 4A–4C). This organization can be compared with previous solid-state nuclear magnetic resonance (SSNMR) results stating that de-esterified pectins are cross-linked by calcium, which increases cellulose-pectin and pectin-pectin contacts, forming a single interconnected network where up to 50% of the cellulose fiber’s surface is contacted by pectins.^43^^,^^44^ Following the amount of meshing throughout a given lamella, we measured a relative decrease in the quantity of meshing in tomograms from ∼0 to 3.5 μm deep in the cell wall (Figure 5H; Video S3). This drop in meshing concentration suggests it is secreted and accumulated at the cell wall-PM interface and in the inner-surface layers and drops out of the deeper, older layers where the tightly packed cellulose layers squeeze the greater part of the meshing out (Figure 5C). This concentrates the meshing at the inner surface of the cell wall, less than ∼1 μm deep. This model of cell wall buildup implies structurally different Z-regions, such that different layers of the wall confer different mechanical properties. This highlights the importance of considering the cell wall as a 3D polylamellate structure.

### Treatments affecting the morphology and quantity of meshing suggest the meshing is HG

The apparent thinning of the cell wall and the measured reduction in meshing abundance upon PL treatment (Figures 6A, 6C, 6D, 6G, S7D, and S7E) suggests that removal of the major pectin, HG, leads to a collapse of the stacked layers, suggesting that HG acts as a filler, intercalating between the cellulose layers (Figures 2B and 2E). This is in line with the latest model of how pectins arrange around the cellulose fibers in the primary cell wall.^44^^,^^45^ The remnants observed in one instance (Figures 6B, 6E, and 6F) could be more complex pectic polysaccharides such as rhamno-galacturonan-II, or the hemicellulosic component xyloglucan, shown to coat the cellulose fibers, possibly bridging them together as we observed in our tomograms (Figure 4C),^28^ or HGs with a higher degree of methylation and therefore little affected by the *Aspergillus niger* pectate lyase.

Additionally, the reticulated networks of purified HGs with an ∼40% methylesterified composition have nearly identical morphology (Figures S9B and S9C) to what was observed *in situ* in native cell walls, adding plausibility to the proposal that HG is the major component of the meshing.

Overall, our results regarding the nature and distribution of the meshing throughout the cell wall suggest that it consists of demethylesterified HGs. This fits well with the current understanding of pectin biosynthesis, where pectins are synthesized in a methylated state in the Golgi apparatus and demethylated *in muro* by endogenous methylesterases^25^^,^^46^^,^^47^ and strongly interact with the cellulose fibers,^43^ participating in the stiffness of the primary cell wall.^44^

### Our measurements of the diameter of the cellulose fibers cannot weight in favor of the 18- or 24-glucan-chain model

Averaging of the cellulose fiber bundle diameter in the three conditions considered was performed in an effort to (1) get an idea of the cross-sectional diameter of cellulose fiber bundles *in situ* and (2) check whether PL or BAPTA treatments could have an effect on bundle thickness, as it has been reported that other wall polysaccharides coat the cellulose fibers.^28^^,^^36^ Given the difficulty of assessing the width of a density line in cryo-ET because of defocus, we opted for the FWHM standardized method (Figure 7D). By this method, we measured fiber bundle diameters ranging from 5.3 to 6.3 nm. PL or BAPTA treatment did not substantially alter the diameter of the cellulose fibers, therefore suggesting that HGs do not coat the cellulose fibers longitudinally. This does not preclude punctual covalent bindings between the HGs and the cellulose fibers, as mentioned in previous models.^48^ Previous reports had measured several hundred fiber bundle diameters in onion walls *in situ* by AFM. The measurements were between 3.5 and 7 nm, which falls within the range of our measurements.^27^ A very recent cryo-ET study on purified cellulose fibers determined their diameters, using a similar method to ours, to be between 4.5 and 6.5 nm, which is very close to our measurements.^49^ Although AFM and cryo-EM studies favor the 18-glucan-chain model of an elementary fiber involving CSCs made of hexamers of trimers,^11^^,^^49^^,^^50^ SSNMR indicates that the elementary cellulose fiber is composed of 24 glucan chains made by hexamers of tetrameric cellulose synthases.^43^ Our measurement, in which averages range from ∼5 to 6 nm with a maximal resolution of 3.8 nm, do not allow a conclusion one way or the other, because we most certainly were only able to measure cellulose fiber bundles comprising at least two elementary fibrils.

### Limitations of study

This work represents, to our knowledge, the first report to use cryo-FIB milling followed by cryo-ET to observe the plant cell wall. Despite the high quality of the data achieved in this study, the throughput of the method is limited by the lengthy milling times and the low survival rate of lamellae from milling to tilt series acquisition. Future work will focus on testing other enzymatic treatments, e.g., those specifically directed toward hemicelluloses like xyloglucan or combinations of enzymes and assess the impact of the degraded component on the structure of the cell wall in 3D and on the diameter of the fiber.

Our study focused on the milling of the periclinal cell wall, as the geometry of their large, flat surfaces (Figures 1E and 1F) made them more amenable to our approach. This does not preclude the feasibility of milling the anticlinal cell walls, even though initial attempts resulted in unstable, very short lamellae unsuitable for cryo-ET. It would be of high interest to visualize the anticlinal cell walls, notably because they constitute interfaces between neighboring cells of the same scale, allowing the visualization of the pectic mid-lamella.^51^ Subsequent studies focusing on the anticlinal cell wall might be enabled by the introduction of more powerful ion sources, which are expected to render milling through thick slabs of material more feasible.^52^ Lastly, while preparing the sample and positioning the cell wall peel on the EM grid, the polarity of the peel relative to the orientation of the onion bulb was not tracked, something to consider for future experiments that would allow comparison of the relative fiber orientations in different scales.

## STAR★Methods

### Key resources table

**Table:** 

REAGENT or RESOURCE	SOURCE	IDENTIFIER
**Chemicals, peptides, and recombinant proteins**		

38% methylesterified citrus pectins – 805 galacturonic acid	Dr Hans-Ulrich - HERBSTREITH & FOX	N/A
Chitosan-OligoSaccharide-Alexa 488 (COS488)	Dr Jozef Mravec	N/A
Pectate Lyase from *Aspergillus*	Megazyme	Cat # E-PCLYAN2
BAPTA buffer	Sigma-Aldrich	Cat # A4926
HEPES buffer	RPI	Cat # H75030-50.0
Tween-20	RPI	Cat # P20370-0.5
CAPS buffer	Sigma-Aldrich	Cat # C2632-100G

**Deposited data**		

Cryo-electron tomogram of non-treated onion cell wall from scale #6 (Figures 2 and 4A–4C)	This paper	EMDB: EMD-26564
Cryo-electron tomogram of non-treated onion cell wall from scale #8 (Figure 3)	This paper	EMDB: EMD-26569
Cryo-electron tomogram of non-treated onion cell wall from scale #2 (Figures 5B, 5D, and 5E)	This paper	EMDB: EMD-26568
Cryo-electron tomogram of purified 38% methylesterified pectins (Figures S6H and S6I)	This paper	EMDB: EMD-26570
Non-treated cellulose fiber average tomogram (Figure 7A)	This paper	EMDB: EMD-26571
BAPTA-treated cellulose fiber average tomogram (Figure 7B)	This paper	EMDB: EMD-26572
PL-treated cellulose fiber average tomogram (Figure 7C)	This paper	EMDB: EMD-26573

**Experimental models: Organisms/strains**		

*Allum cepa* (White onion)	Pavilions supermarket (845 E California Blvd, Pasadena, CA 91106)	N/A

**Software and algorithms**		

EMAN2 software	Chen et al.^2^	https://blake.bcm.edu/emanwiki/EMAN2
Amira software	FEI, Thermo Fisher Scientific, Rigort et al.^32^	https://www.thermofisher.com/us/en/home/electron-microscopy/products/software-em-3d-vis/amira-software.html
IMOD	Kremer et al.^3^	https://bio3d.colorado.edu/imod/
SerialEM	Mastronarde 2003	https://bio3d.colorado.edu/
Gatan DM3	N/A	https://www.gatan.com/products/tem-analysis/gatan-microscopy-suite-software
ImageJ	N/A	https://imagej.nih.gov/ij/download.html
R	N/A	https://cran.r-project.org/index.html
R studio	N/A	https://www.rstudio.com/
*MeshingSubtract* bash script	This paper	10.13140/RG.2.2.17904.53764

**Other**		

Quantifoil London Finder NH2 R2/2 Copper 200 mesh + extra thick carbon	EMS	Cat #LFH2100CR2
Vitrobot Mark IV	FEI, Thermo-Fisher	https://www.thermofisher.com/us/en/home/electron-microscopy/products/sample-preparation-equipment-em/vitrobot-system.html
FIB autogrids	FEI, Thermo-Fisher	Cat #1205101
Zeiss LSM 880 Airyscan	Zeiss	Out-of-production; https://www.zeiss.com/microscopy/int/products/confocal-microscopes/lsm-980.html?vaURL=www.zeiss.com/lsm880
Versa 3D DualBeam FIB-SEM	FEI	Out-of-production
Quorum transfer system	PP3000T	https://www.quorumtech.com/products/cryo-sem-preparation-systems/
Gatan K3 summit camera with post-column energy filter	Gatan	https://www.gatan.com/products/tem-imaging-spectroscopy/k3-cameras
300 kV Titan Krios microscope	FEI, Thermofisher	https://www.thermofisher.com/us/en/home/electron-microscopy/products/transmission-electron-microscopes.html

### Resource availability

#### Lead contact

Further information and requests for resources and reagents should be directed to and will be fulfilled by the lead contact, Elliot Meyerowitz (meyerow@caltech.edu).

#### Materials availability

This study did not generate new unique reagents

### Experimental model and subject details

#### *Allum cepa* (white onion)

White onions were purchased the day of or the day prior to the experiments at the local Pavilions supermarket (845 E California Blvd, Pasadena, CA 91106). Peels at the various concentric scales used throughout this work were generated as described in Kafle et al.^29^ and Durachko et al.^30^ Briefly, the scales were sliced longitudinally with a sharp knife or razor blade. Then the middle of each slice, where the width is more or less constant, a ∼4 cm long piece was cut out. An incision was made with a sharp razor blade approximately 1 cm away from the edge, creating “handles”. Then these handles were used to pull apart the epidermal layer away from the parenchyma of the scale. This resulted in peels about 1 cm in width and 2-3 cm in length. These peels were incubated for at least 20 min in HEPES buffer (20 mM HEPES, RPI H75030-50.0; 0.1% Tween-20, RPI P20370-0.5; pH 6.8 with KOH) and remained in it until freezing. Before freezing, each cell wall peel was mounted between slide and coverslip and screened with a table-top microscope equipped with phase-contrast to ensure that the peel had a homogenous surface of cleanly ruptured cells where only the cell wall remained. Phase-contrast allowed visualization of the remaining floppy, jagged-looking anticlinal cell walls, indicating that peeling of the cell wall was successful.

### Method details

#### Quantification of the aspect ratios of epidermal cells by light microscopy

A large montage of the epidermal cell wall peels was acquired by light microscopy using a Nikon 90i epifluorescence microscope. These maps were segmented with ImageJ with the following method: i) out-of-focus cells and folded-over peels were masked out manually to avoid distorted cell segmentations using the polygon selection tool of imageJ and deleting the selected areas. ii) A binary mask was applied on the montages in order to select the outline of the cells, and the resulting mask was gaussian-filtered (2 pixel) and skeletonized. iii) The “Analyze particles” tool was used to detect closed cells and calculate their aspect ratio.

#### Enzymatic treatments and staining

Pectate lyase from *Aspergillus* (Megazyme, 180 U/mg, Cat # E-PCLYAN2) at 4.7U/mL (8uL of stock solution in 5mL of 50 mM CAPS buffer, Sigma-Aldrich A4926; pH 10)^10^ and BAPTA calcium chelation at 2mM (Sigma Aldrich – Cat # A4926) treatments were performed on cell wall peels generated as described above. Treatments were carried out for 3 hours and 10 min, respectively, on the peels.

To screen for the effectivity of the treatments prior to vitrification, staining of non-treated, BAPTA- or PL- treated onion peels by a homogalacturonan-specific probe, Chitosan OligoSaccharide coupled with Alexa-488 (COS488) (Figure S6B) was performed based on a protocol provided by Jozef Mravec (personal communication): 1:1000 dilution from the mother solution kindly provided by Jozef Mravec (kept at -20C wrapped in foil) in 50 mM MES buffer pH 5.8 for 15 min. Peels were then washed with DI water 3 consecutive times before being mounted between a slide and coverslip and then screened by confocal laser scanning microscopy.^53^

#### Purified pectin preparation

Citrus-derived high homogalacturonan content purified pectins were kindly provided by Professor Hans-Ulrich, from Herbstreith & Fox (https://www.herbstreith-fox.de/en/): Pectin Classic CU 701 (38% methyl-esterification, 89% galacturonic acid content). 10mL of 2.5% (w/v) aqueous pectic solutions were made (pH 3.4 according to manufacturer’s MSDS sheet). Serial dilutions at 0.25% and 0.125% were then prepared from the 2.5% solution.

#### Plunge-freezing

##### Onion cell wall peels

The cell wall peels previously incubated in HEPES buffer were laid on a slide with a drop of HEPES buffer to keep the cell wall hydrated. After incubation in HEPES buffer, the peels were mounted in a drop of HEPES on a slide. A tangential light was shined at the peel to increase visibility. If possible, a magnifying glass affixed on a support can be used. Small rectangular pieces (∼2 x 3 mm) were cut out of the cell wall peel with a sharp razor blade and carefully dragged on the carbonated side of glow-discharged (15mA – 1min) Quantifoil R2/2 NH2 Cu EM grids (EMSdiasum). Plunge freezing was performed with a 60/40 ratio ethane/propane mix and an FEI Vitrobot Mark IV (Thermo Fisher). Humidity was set at 50%, temperature at 20°C. Grids were first manually backblotted for 6 s in order to attach the cell wall peel firmly to the carbon, followed by two autoblottings (front and back) 5 s, maximal blot force (25) and a drain time of 3 s.

##### Purified pectins

5uL of 2.5%, 0.25% and 0.125% purified pectin was pipetted onto Quantifoil R2/2 NH2 Cu EM grids (EMSdiasum) and the grids were plunge frozen at 100% humidity, 20C with a blot time of 4 s, a medium blot force of 10 and a drain time of 1 s.

#### Cryo-FIB milling

During the grid clipping stage, prior to milling, orientation of the peel is important, so the long side of the rectangle was positioned parallel to the notch in autogrid holders (Thermo Fisher) machined with a notch. Like this, the shorter side of the anticlinal cell walls are orthogonal to the FIB beam leading to less obstructed areas of the periclinal cell wall and thus more potential FIB-milling targets. Autogrids were placed in a custom-built shuttle and inserted into a Versa 3D dual-beam FIB/SEM microscope with a field emission gun (FEG) (FEI) equipped with a PP3000T cryo-transfer apparatus (Quorum Technologies). They were maintained at -175°C at all times by a custom-built cryo-stage.^54^ To reduce sample charging and protect the sample from curtaining during milling, the grids were sputter-coated with platinum at 15mA for 60 s. Thin lamellae were generated with the gallium ion beam at 30 kV at angles ranging from 10 to 17°. Rough milling was done at high currents, ranging from 0.3 nA to 100 pA, until the lamellae measured 1 μm in thickness under the FIB view. The current was then progressively brought down to 10 pA for the final milling steps until the measured thickness was between 100 and 200 nm Final polishing by tilting the sample 0.5 to 1° to homogenize the lamella thickness was also done at 10 pA. During the whole procedure, imaging with the SEM beam was done at 5 kV and 13 pA. SEM overviews were used to precisely outline and measure the respective aspect ratios (width vs. length) of the milled cells. When In-chamber Gas Injection System (GIS) Pt coating was performed, the needle was set at 26C and flushed for ∼10s before injection onto the onion peel. The injection was performed for ∼5 s at a distance of +2 mm from eucentric height.

#### Confocal microscopy

Confocal analysis of the onion cell wall peels stained with the COS488 stain was performed on a ZEISS LSM880 equipped with Airy Scan and a GaAsP detector. Magnification used was 40x (C-Apochromat 40x/1.2 W Korr M27). Channel settings were set as follows and kept constant throughout the conditions screened: For the Alexa 488 channel the excitation Ar laser (488 nm) was set to 0.3% power, the gain was set to ∼700 and pinhole was set to ∼10 AU with a pixel dwell time of ∼2 μs. A GaAsP detector was used, and the detection range was set from 499 to 630 and the 488 main beam splitter was used. Trans-channel was set with a gain of ∼450. Z-stacks were acquired with the optimal Z-step defined by the software, 1.55 μm.

#### Electron-cryotomography

Tilt-series acquisition was performed on a Titan Krios (Thermo Fisher) equipped with a GIF post-column energy filter (Gatan) and a K3 direct detector 6k x 4k (Gatan). Data acquisition was controlled via SerialEM^55^ with a 3° tilt increment for a total range of ±60° or ±50°, a defocus of -10 μm, and a total dose up to 80 e^-^/A^2^. No pre-tilt was applied, and a bi-directional tilt scheme was used. Tilt series were then aligned via patch tracking with the IMOD package^56^ reconstructed using weighted back projection and the SIRT-like filter set to 15 iterations.

#### Mapping out tomograms on the milled cells

Orientation of the grid is lost during the transfer from the cryo-SEM chamber to the cryo-TEM autoloader. The grid can be rotated and/or flipped over. This necessitated correlating the orientations found in cryo-SEM and the cryo-TEM data. We, therefore, used the high-resolution montage maps of the lamellae as the reference where the different fields of view of the tomograms can be seen. Using Adobe Illustrator, the high-resolution TEM montages were correlated with the TEM grid montages and the cryo-SEM-overviews of the lamellae. The latter are flipped and rotated if needed to fit the final orientation in the TEM used for data collection. Finally, the angle between the X-axis of the tomograms and the long axis of the cell was registered. This ensured the precise knowledge of the long axis of the milled cell within each tomogram, which in turn allowed the extraction of biologically relevant numbers.

Depth of the tomograms in the cell wall was computed using the nominal milling angle as the inclination and the projected distance d between the leading edge of the lamella (top of the lamella, identified by the presence of platinum) and the center of the ROIs for tilt series acquisition.

#### Sub-tomogram averaging and cross-sectional measurements

Sub-tomogram extraction, alignment, and averaging were performed using the Dynamo software package.^57^ Initial orientations and positions of cellulose fibers segments were determined using geometrical tools for particle picking in Dynamo.^58^ Regions of the filaments with minimal bending and overlapping were traced in 4x binned tomograms. Centers of the particles were placed every ∼70 Å along the filament. Final sub-volumes were extracted from 2x binned tomograms with a final pixel size of 6.7 Å and 40x40x40 box size. The total number of sub-tomograms ranged from 750 to 1100 for all three datasets. Initial reference for particle alignment was generated by averaging segments with azimuth randomized orientations. Iterative alignment and averaging procedures were performed according to gold-standard in Dynamo. A loose cylindrical mask was applied for the alignment step. The final mask corrected FSC was estimated in RELION3 using a soft-edge mask (Figure 7E).^59^

#### Measurement of the cross-sectional diameter

The *sideview-profile*-*average* script^60^ was used by tracing an open contour in the middle of the fiber in 3dmod. The following parameters were used: step 1 pixel, length 30 pixels, and thickness 10 pixels. The output json files were imported into R. The average pixel intensities were double normalized relative to the lowest and highest pixel values in each profile to compare curves between conditions. The Full-Width-at-Half-Maximum (FWHM) was used by measuring the width of the gaussian bell at 0.5 relative pixel intensity.

#### Tomogram segmentation

##### Fiber segmentation

Segmentation was performed on filtered tomograms with the default parameters of EMAN2 (low-pass gaussian cutoff of 0.25 and high-pass gaussian cutoff of 5px) and Convolutional Neural Networks (CNN)^31^ were used to recognize the fibers in the tomograms (Figures S1A–S1C). Training was performed on several tomograms by boxing ∼20 positive examples and ∼100 negative examples. The positive examples were precisely segmented using a graphical tablet (*Wacom Cintiq 21uX*) and the CNNs were trained with the default parameters except for the learn rate that was increased in some instances to 0.001 instead of the default 0.0001. The outcome of the trained CNN was checked on the boxed particles and if satisfactory the CNN was applied on the tomogram. Eventually, a second round of training was performed with additional boxes from another tomogram from the same dataset or on itself. The resulting CNN map was then carefully examined versus the filtered tomogram to ensure they agreed, and segmentation was specific to the fibers. For tomograms acquired over the same session on the same lamellae, the same CNN was able to generalize well and segment accurately. Tomograms from different datasets and different lamellae usually required retraining a CNN.

Satisfactory CNN segmented volumes were then transferred into *Amira* (Thermo Fisher) to perform template matching fiber tracing with the *TraceX Amira* plugin^32^ (Figures S1D–S1F) in order to model the fibers as a set of connected nodes. To be able to optimize parameters, we reduced the processing time by binning twice (binning 8 total) the CNN maps. The first step, *Cylinder Correlation*, was performed with the following starting parameters: cylinder length of 50 pixels, an angular sampling of 5, and missing wedge compensation was toggled. The diameter of the template (outer cylinder radius) was set to closely match the apparent diameter of the fibers in the tomogram, usually 4 pixels. As advised in the Amira user guide section 3.8 on the *XTracing Extension*, the mask cylinder radius was set to 125% of the outer cylinder radius. The outcome was visually checked to see if the fibers were detected correctly and not too many artefacts were generated. Parameters were slightly modified one-by-one if needed to improve the output. The subsequent step, *Trace Correlation Lines* was performed with the following nominal parameters: minimal line length 60 pixels, direction coefficient 0.3, and minimal distance of 2-times outer-cylinder diameter used previously. Minimum seed correlation and minimum correlation are tomogram-dependent parameters. These values were defined on the correlation field by defining the reasonable correlation value range. The minimum seed correlation and minimum continuation quality are the upper and lower limits of the range, respectively. For the search cone, length was set to 80, angle to 37°, and minimal step size was 10%. The outcome was visually checked to see if the fibers were being traced correctly. To do so, we used the *Spatial Graph View* function and checked for artificial fiber trackings. Parameters were modified if needed to enhance fiber detection and reduce false discoveries. Because of the inherent nature of the signal of cryo-ET volumes and their CNN maps, punctate signals would generate and propagate artefactual vertical (parallel to the Z-dimension) lines. These were first selected by using a Tensor XZ and Tensor ZZ visualizer in the *Spatial Graph View* window and identifying the appropriate thresholds. After the coordinates of all fibers were extracted as a.xml file, fiber tracks with values above/below the thresholds were trimmed out.

##### Meshing segmentation

The method to output the CNN maps recognizing the meshing is identical to the one used to segment the fibers. We were unable to generate a CNN that could specifically pick up the meshing. Instead, we resorted to training CNNs that could recognize all features in the tomograms and then subtracted this density map with the one generated from the fiber-trained CNN (Figure S2). This allowed isolation of identified features that were not fibers, assuming that everything that is not fiber belongs to the “meshing” feature. This was done using a custom script called *MeshingSubtract* (10.13140/RG.2.2.17904.53764) that relies on IMOD and bash commands. First, the fiber-CNN map was thresholded. The level of the threshold is chosen in order to mask the fibers as accurately as possible. This mask is then subtracted from the meshing-CNN map to create the subtracted meshing map.

To quantify the volume occupancy of these two features, the *imodauto* command was used on the fiber and subtracted meshing. A threshold of 0 was used on the masked fiber-CNN. For the subtracted meshing map, the threshold was chosen in order to segment as accurately as possible the meshing by comparing with the low-pass filtered tomogram. Both resulting segmentations were joined using the *imodjoin* command and the *imodinfo* command was used to compute the volume occupancy of each segmented feature (the value taken was the cylinder volume).

### Quantification and statistical analysis

#### Data extraction

Point (containing only point number, x-, y- and z-coordinates) and segment data (containing only point numbers) from the Amira-Avizo (Thermo Fisher) software was exported as tab-delimited files. The reformat_amira_output.m,^61^ available from https://schurlab.ist.ac.at/downloads/ was used to convert these files into IMOD formatted tab-delimited text files, which were then further analyzed using custom scripts in python.

First, all contours with an out-of-plane angle of larger than 70 degrees were removed, as those did not correspond to fibers but rather to tomogram reconstruction artifacts. For each model, the long axis of the cell was accurately determined as detailed above. Then the model was rotated around the y-axis to reposition the volume according to the angle applied during the milling step and lost during the volume flattening occurring during tomogram reconstruction.

To overcome the uneven spacing of points on contours exported from Amira, fiber contours were interpolated using cubic splines resulting in a sampling rate of 1nm along the length of the fiber. From these reoriented volumes in the cell wall, fiber length, radius of curvature, slope of the fiber, clockwise angle of the fiber relative to the cell’s long axis,

The length of individual fibers was calculated as the sum of distances between neighboring points along its run:

$$length=\sum_{i=1}^{n-1}distance\left(point_{i},point_{i+1}\right)$$

With n being the number of points of the given contour representing the fiber.

The curvature radius was calculated by averaging the local curvature radii over all triples of neighboring points within a given contour. For this the reciprocal relationship between the Menger curvature and the curvature radius was employed:

$$radius\;of\;curvature=\sum_{i=1}^{n-2}\frac{distance\left(point_{i},point_{i+1}\right)*distance\left(point_{i+1},point_{i+2}\right)*distance\left(point_{i},point_{i+2}\right)}{4*area\left(point_{i},point_{i+1},point_{i+2}\right)}$$

With n being the number of points of the given contour representing the fiber.

Prior to calculating local slopes along the run of a fiber, the sequence of the points was, if necessary, adjusted so that the first point of the contour would have a lower y-coordinate than the last point of the contour. This was done to establish a common direction for all fibers within a tomogram. To calculate the slope between two neighboring points on a contour representing a fiber the difference between their z-coordinate is divided by their distance in the xy-plane:

$$slope=\frac{value_{z}\left(point_{i+1}\right)-value_{z}\left(point_{i}\right)}{distance_{xy}\left(point_{i},\;point_{i+1}\right)}$$

With value_z_(point) extracting the z-coordinate of a given point and distance_xz_(point, point) calculating the distance between two points only considering x- and y-coordinates.

For calculating the average z-height of a fiber, the z-coordinates of all points in the respective contour were averaged.

For calculating the angle between a fiber and the long axis of the cell, the orientation of the fiber was approximated by a vector pointing from its end with the lower y-coordinate value to its end with the higher y-coordinate value. The vector representing the long axis of the cell was calculated from the orientation of the cell on the grid and the rotations applied during tomogram reconstruction.

$$Angle\;to\;long\;axis\;of\;the\;cell=degree(arctan2\left({\vec{c}}_{x}*{\vec{f}}_{y}-{\vec{c}}_{y}*{\vec{f}}_{x},{\vec{c}}_{x}*{\vec{f}}_{x}+{\vec{c}}_{y}*{\vec{f}}_{y}\right)$$

With x and y representing the x and y scalars of the vectors of the long axis of the cell $\left(\vec{c}\right)$ or the fiber $\left(\vec{f}\right)$, respectively. The resulting angles in radians were then transformed to degrees as depicted in the figures.

The custom scripts applying these operations can be further found in Dimchev et al.^61^

#### Data analysis and visualization

All the data analysis, data exploration and statistical analysis was performed with R using the *here, scales*, and *tidyverse* libraries. All plots made with *ggplot2* and all results are expressed as mean ± standard deviation.

##### Quantification of the meshing/fiber ratio

This data is found in Figures 2F, 4G, 4M, 5H, and 6G.

To quantify the volume occupancy of these two features, the *imodauto* command was used on the fiber and subtracted meshing. A threshold of 0 was used on the masked fiber-CNN. For the subtracted meshing map, the threshold was chosen in order to segment as accurately as possible the meshing by comparing with the low-pass filtered tomogram. Both resulting segmentations were joined using the *imodjoin* command and the *imodinfo* command was used to compute the volume occupancy of each segmented feature (the value taken was the cylinder volume). The values were consigned in a table, the meshing/fiber volume occupancy ratio was computed and was consequently used for data analysis.

For the histograms (Figures 2F, 4G, and 4M), fiber and meshing volume occupancies were normalized relative to the total volume occupancy (fiber volume occupancy + meshing volume occupancy).

For the graphs (Figures 5H and 6G), the non-normalized meshing/fiber volume occupancy ratio was plotted. The inset in Figure 6H uses the same non-normalized volume occupancy ratios were used.

##### Quantification of the orientation, average height, slope and radius of curvature of each fiber

This data is found in Figures 3C, 3D, 6H, S3, S4A, S4B, S5B, and S5C. Here *ggExtra* library was used for Figure S3

Each fiber taken into account appears as one line in a data frame, holding several identifiers such as the orientation value, the average Z-height, radius of curvature, slope, tomogram and its depth in the cell wall (Figure 1J), scale where lamella was milled, aspect ratio of the cell milled, treatment applied to the peel pre-freezing, etc.

For the distribution plots (Figures 3C and 6H), histograms (*ggplot2 > geom_histogram*) with orientation as the x component, with a bin of 5° and density plots (*ggplot2 > geom_density*) were overlapped with each other.

For the scatterplots (Figures 3D and S3), each point represents a fiber where the x coordinate is its average Z height and its y coordinate is its orientation (*ggplot2 > geom_point and geom_density_2d*). ggExtra > ggMarginal was used to add the histogram on the side of the scatterplot (Figure S3).

For the violin plots against the depth of the tomogram was done (Figure S4A) using depth of the tomogram as the x component and orientation as the y component and scaling according to “width” (*ggplot2 > geom_violin*). Plots were generated separately for each scale where the lamella was milled. For violin plots against aspect ratio of the milled cell (Figure S4B), same parameters were used to generate the violin plots but the data was pooled according to the aspect ratio (x axis) instead of the depth.

To determine the modal values in each tomogram, necessary for computing the means (Figures 3C and 6H), the distributions of the orientations were fitted using mixed models (*dipTest* and *mixtools* libraries).

##### Quantification of the Full Width at Half Maximum (FWHM) of fiber cross-sections

This data is found in Figure 7D.

JSON files generated by the *sideview-profile* script are imported using the *jsonlite* library. To increase accuracy, 0.5 pixel interpolation was performed and the data was double normalized relative to min and max values of each curve in order to allow comparison between the conditions.

##### Quantification of the aspect ratio of onion cells screened by light microscopy

This data is found in Figures S4C and S4D

For the distribution plots (Figures S4C and S4D), histograms (*ggplot2 > geom_histogram*) with orientation as the x component, with a bin of 0.05 and density plots (*ggplot2 > geom_density*) were overlapped with each other. Grouping was done either according to the onion used (Figure S4C) or according to the scale # used (Figure S4D, which pools data from all onions together).

##### Quantification of the normalized fluorescence intensity of the COS488-stained onion cell wall

This data is found in Figure S6C.

ROIs were traced to fit the entire periclinal cell wall on the maximal projections of the stacks and the average intensity was computed. Minimal and maximal pixel values over the whole image were used to double normalized the mean intensities. Plotting of these mean intensities, grouped by treatment condition was done (*ggplot2 > boxplot*).

## Data Availability

The tilt series, tomograms,.mdoc and.rawtlt used for the data analysis are available upon request. Representative tomograms have been deposited in the Electron Microscopy Data Bank (EMDB) under accession codes EMDB: EMD-26564, EMD-26569, EMD-26568, and EMD-26570. The three cellulose fiber averages have been deposited in EMDB under accession codes EMD-26571, EMD-26572, and EMD-26573. See key resources table for individual description. The bash script used to generate the meshing subtracted maps and quantify the fiber/meshing ratios was deposited on ResearchGate (https://doi.org/10.13140/RG.2.2.17904.53764). DOIs are lister in the key resources table.

## Supplemental information

Document S1. Figures S1–S7 and Table S1

Document S2. Article plus supplemental information
