# Supplementary material for: Cryo-electron tomography of the onion cell wall shows bimodally oriented cellulose fibers and reticulated homogalacturonan networks
Source: Curr Biol. 2022 Jun 6;32(11):2375–2389.e6. doi: 10.1016/j.cub.2022.04.024 (PMC9240970; doi:10.1016/j.cub.2022.04.024)
Supplement: Document S1. Figures S1–S7 and Table S1 [file mmc1.pdf]

Current Biology, Volume *32*

Supplemental Information

Cryo-electron tomography of the onion cell wall shows bimodally oriented cellulose fibers and reticulated homogalacturonan networks

William J. Nicolas, Florian Fäßler, Przemysław Dutka, Florian K.M. Schur, Grant Jensen, and Elliot Meyerowitz

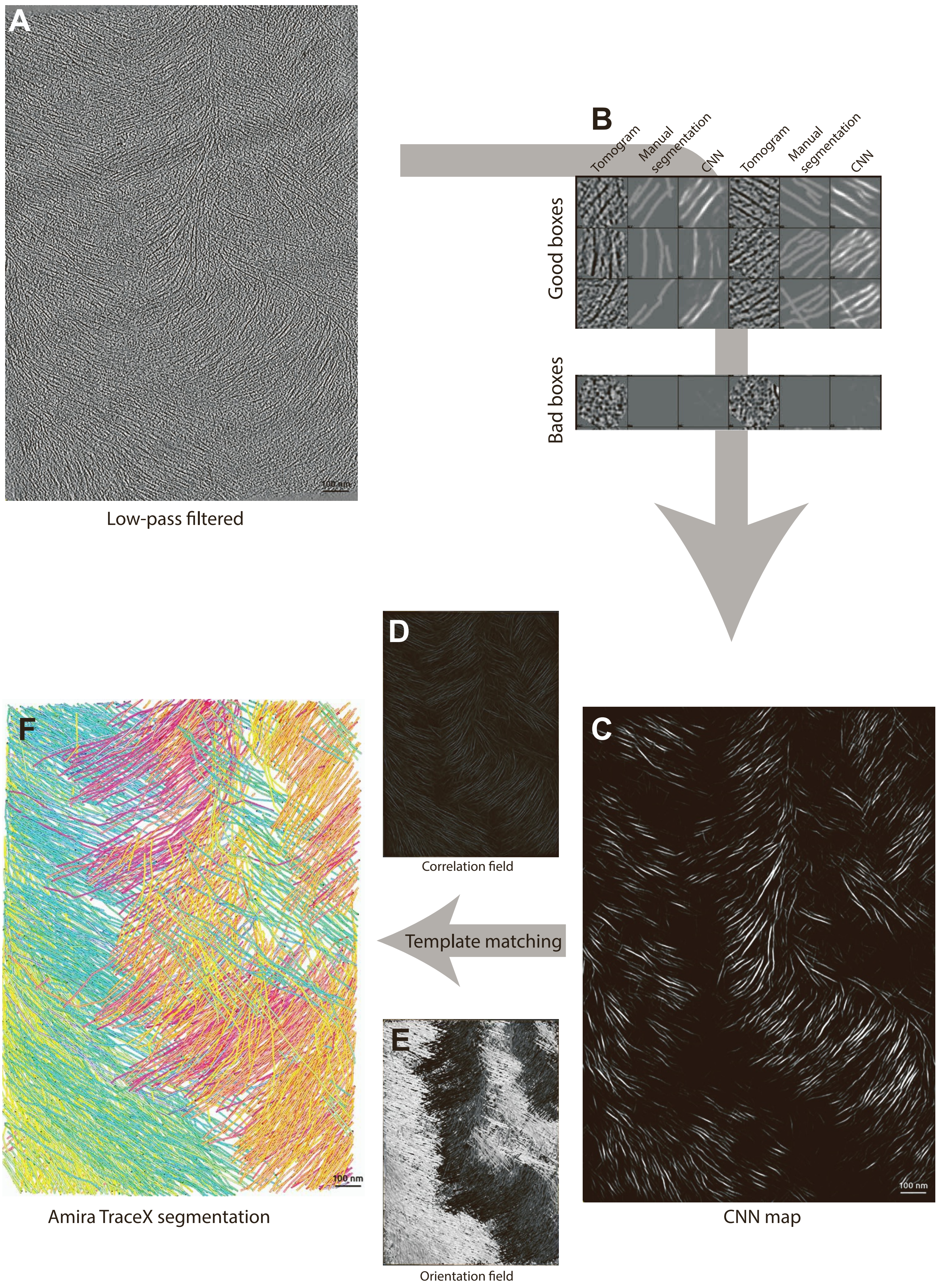
A
100 nm
Low-pass filtered
B
Tomogram
Manual segmentation
CNN
Tomogram
Manual segmentation
CNN
Good boxes
Bad boxes
C
100 nm
CNN map
D
Correlation field
Template matching
E
Orientation field
F
100 nm
Amira TraceX segmentation

Figure S1 | Convolutional Neural Networks and Template matching based segmentation of the tomograms. Related to Figure 2 and STAR methods.
(**A**) Example tomographic slice of a low-pass filtered tomogram. (**B**) Example training boxes of cellulose fibers. From left to right, the columns are the boxed sub-tomograms, the manual segmentation provided for the training, and the CNN segmentation. (**C**) EMAN2-CNN segmented tomogram. (**D, E**) Correlation and orientation field outputs generated by Amira during the template matching step with the template being a 50 pixels long, 4 pixels wide outer-cylinder diameter. (**F**) Final segmentation of the segmented fibers.

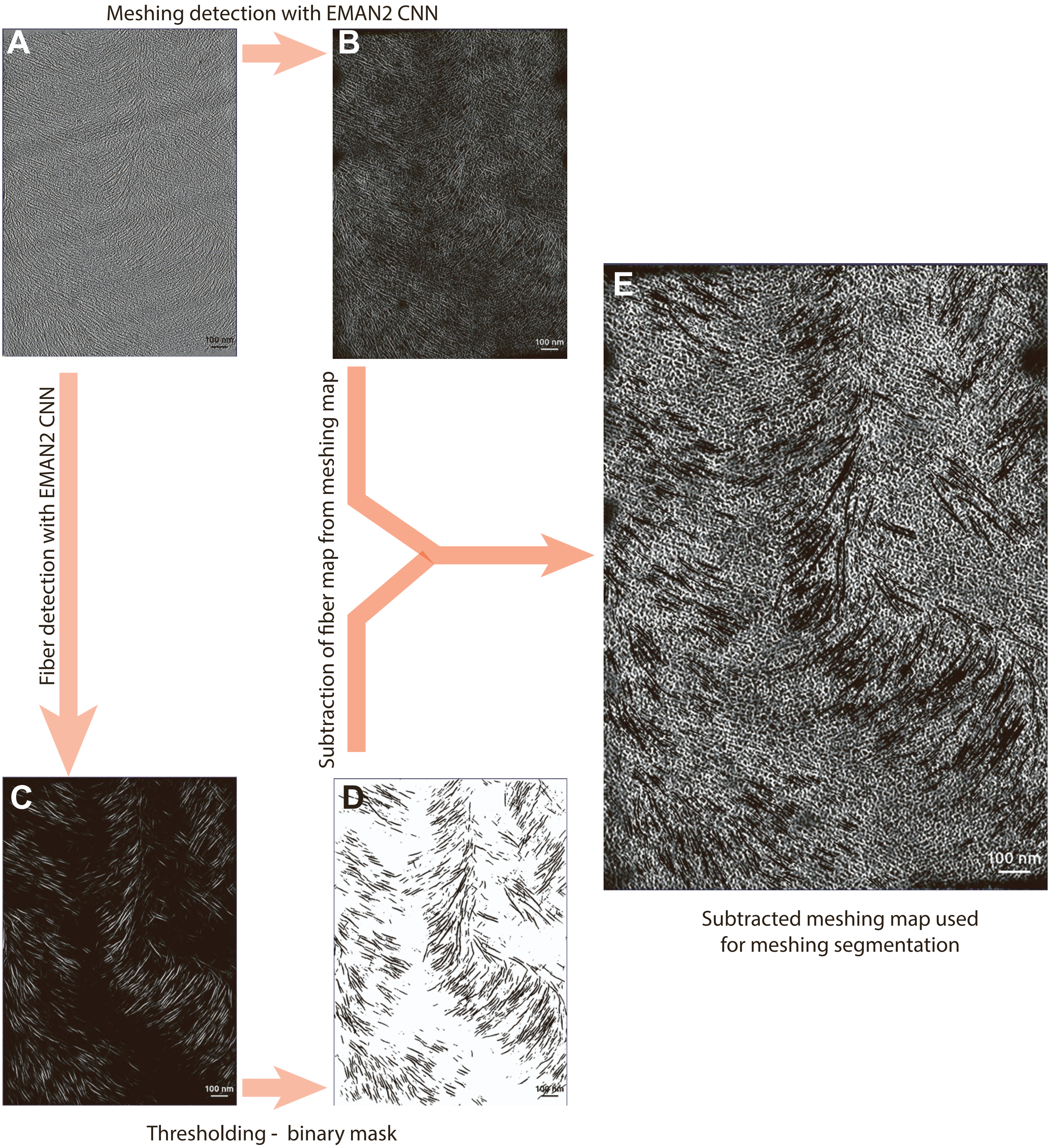
Meshing detection with EMAN2 CNN
A
B
100 nm
100 nm
Fiber detection with EMAN2 CNN
Subtraction of fiber map from meshing map
E
100 nm
C
D
100 nm
100 nm
Thresholding - binary mask
Subtracted meshing map used
for meshing segmentation

Figure S2 | Segmentation of the meshing. Related to Figure 2 and STAR methods.
(**A**) Tomographic slice of a low-pass filtered tomogram. (**B**) Tomogram segmented by a CNN trained to recognize the meshing. (**C**) Tomogram segmented by a CNN trained to recognize the fibers. (**D**) Masked fiber-segmented tomogram. (**E**) Meshing-segmented tomogram (B) subtracted by the thresholded fiber-segmented tomogram (D).

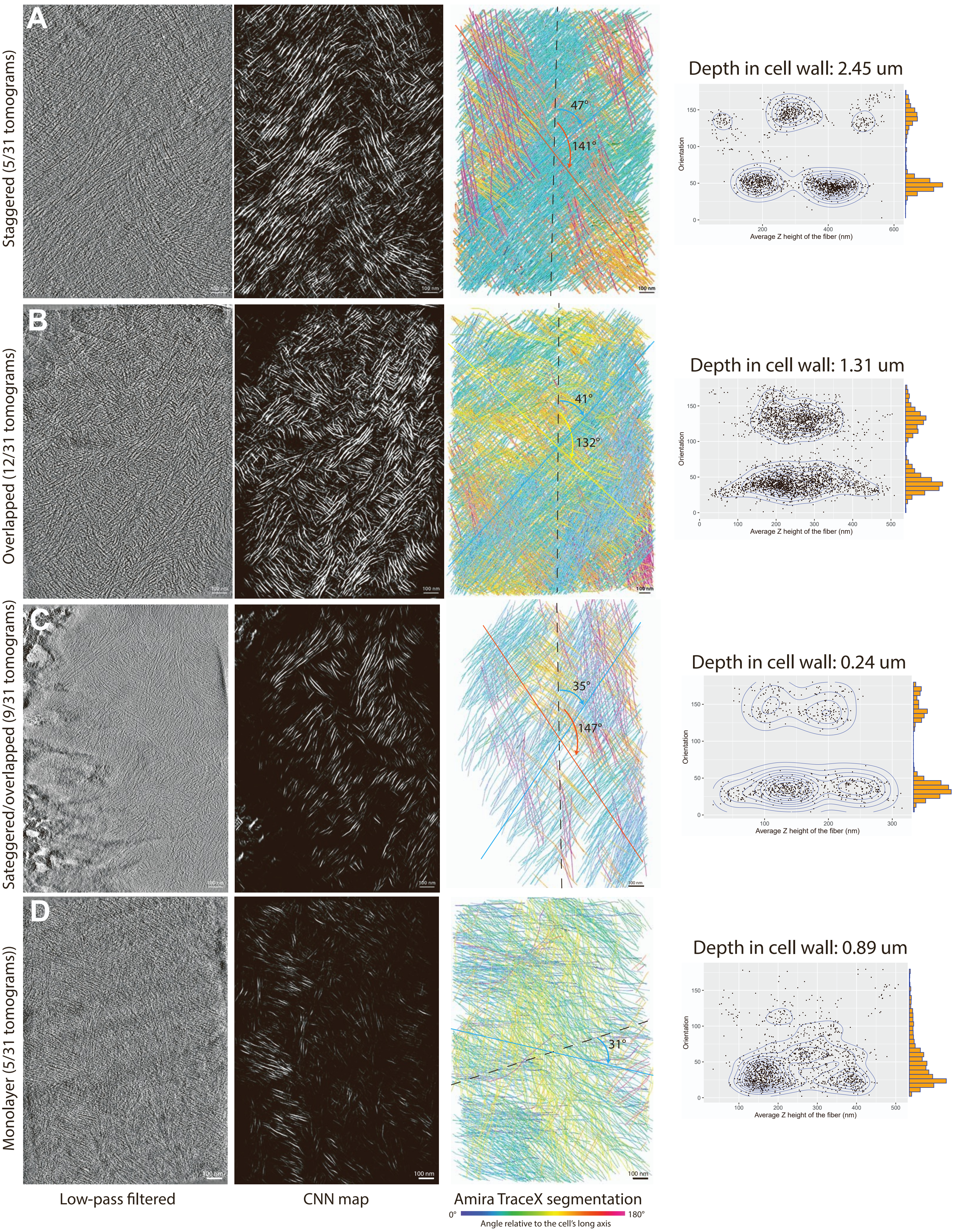
A
Staggered (5/31 tomograms)
47°
141°
Depth in cell wall: 2.45 um
Orientation
Average Z height of the fiber (nm)
B
Overlapped (12/31 tomograms)
41°
132°
Depth in cell wall: 1.31 um
Orientation
Average Z height of the fiber (nm)
C
Sateggered/overlapped (9/31 tomograms)
35°
147°
Depth in cell wall: 0.24 um
Orientation
Average Z height of the fiber (nm)
D
Monolayer (5/31 tomograms))
31°
Depth in cell wall: 0.89 um
Orientation
Average Z height of the fiber (nm)
100 nm
Low-pass filtered
CNN map
Amira TraceX segmentation
0°
180°
Angle relative to the cell's long axis

Figure S3| The different fiber orientation layering patterns. Related to Figure 3.

From left to right: The low-pass filtered tomograms, the CNN segmented tomograms, the Amira segmented volumes displaying the cell's long axis (black dashed line) and the main modes, and the scatterplot of the orientation of the fiber as a function of its average Z-height. (**A**) The staggered pattern characterized by clean successive -/+ 45° cellulose fiber layers, with clearly defined clusters in the scatterplots. (**B**) The overlapped pattern where the two -/+ 45° are intercalated with each other. (**C**) The overlapped-staggered pattern, similar to (A) but the scatterplot shows an overlapping cluster. (**D**) The monolayer pattern showing only one main mode but a very scattered cluster, as shown in the scatterplots.

Effect of depth in the cell wall on the angular distribution of fibers

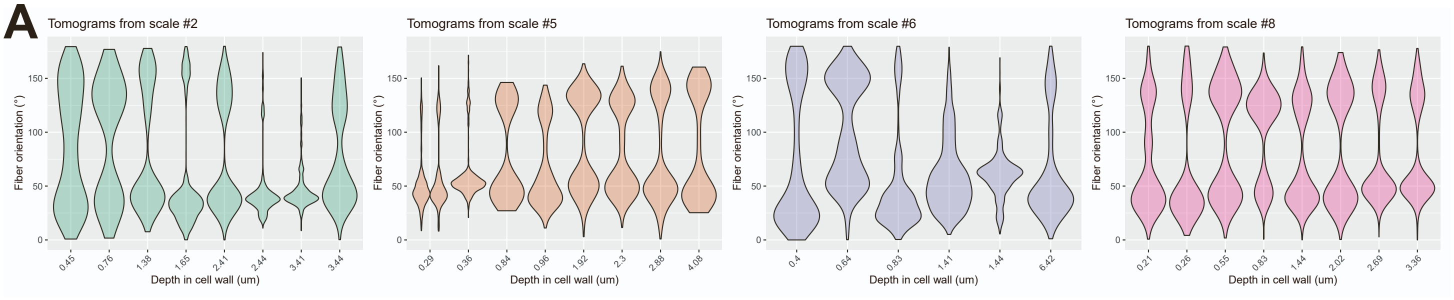

Effect of aspect ratio of the cell on the angular distribution of fibers

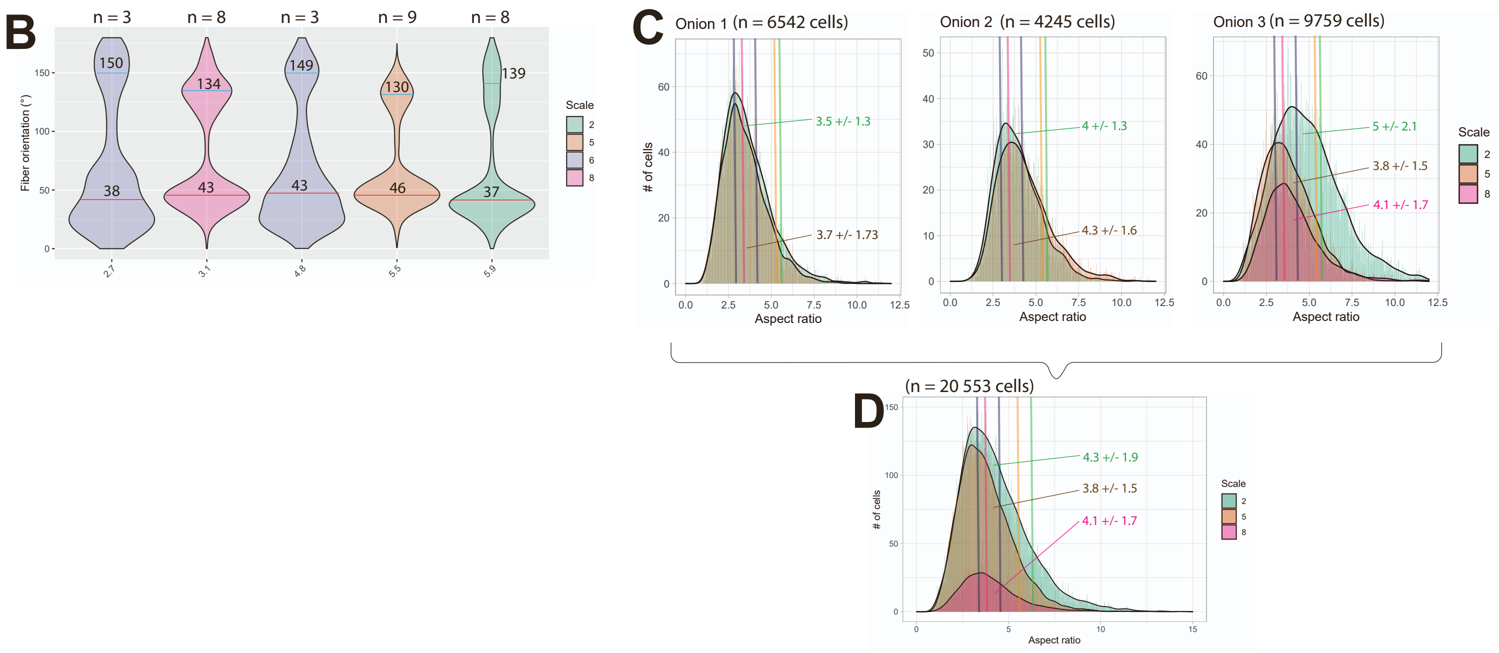

Figure S4 | The bimodal angular pattern is found in cells of all aspect ratios and at all depths. Relate to Figure 3.

(**A**) Violin plots for each scale (scale #2, 5, 6 and 8, from left to right), showing the distribution of the fiber angles as a function of the depth of the tomographic volume in the milled cell wall. One violin represents one tomogram. (**B**) Violin plots for each milled cell, showing the distribution of the fiber angles as a function of the aspect ratio of the milled cell. The violins are color-coded per scale and the X-axis shows the aspect ratios of the milled cells. (**C**) Distribution of the aspect ratios of cells screened by light microscopy (see methods) in 3 different onions. Colored vertical lines represent the aspect ratios of the milled cells in (B) and show that the milled cells fall within the range of aspect ratios of their respective scales. (**D**) Same as (C) but all cells from the three onions were pooled.

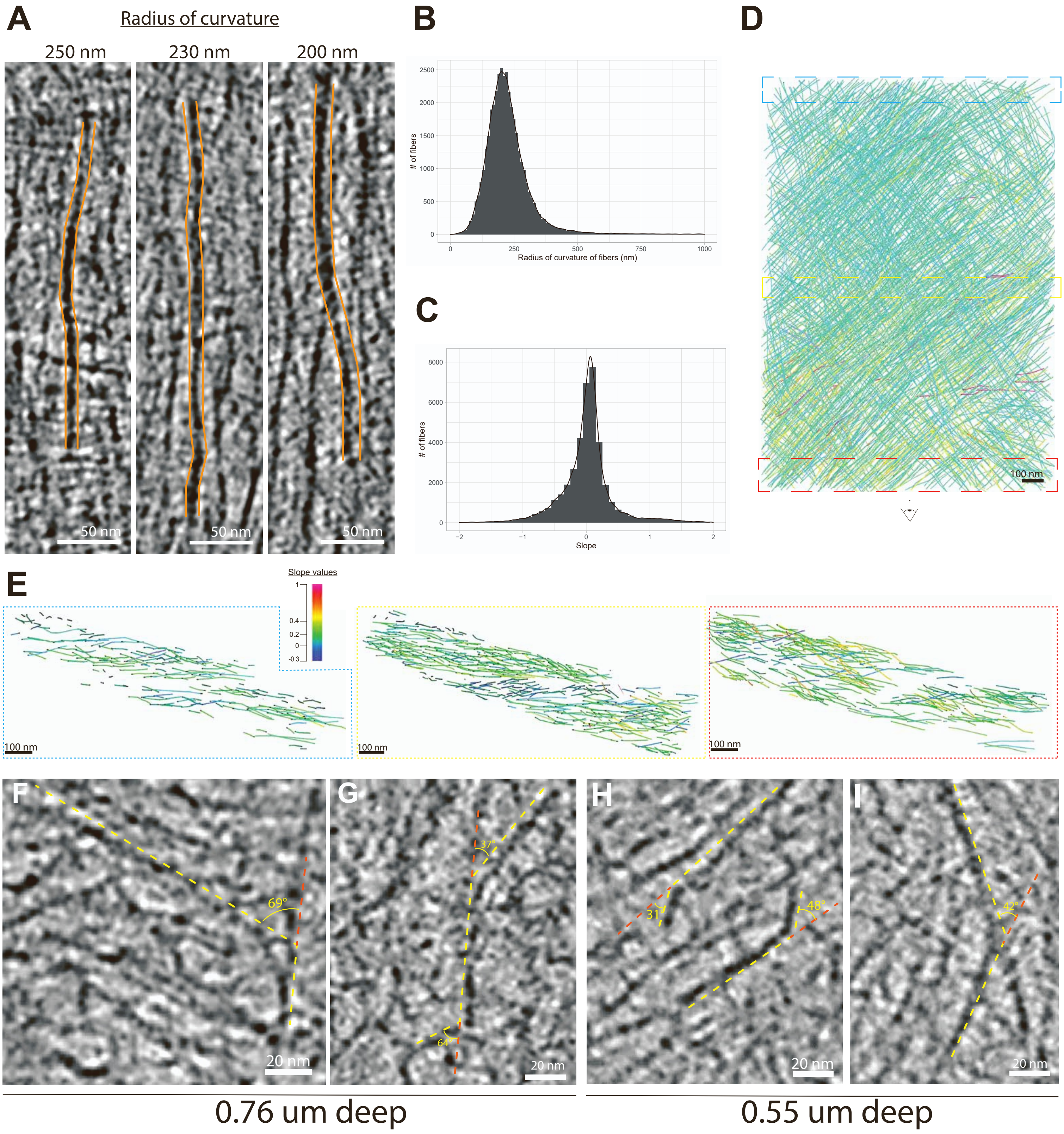
A
Radius of curvature
250 nm
230 nm
200 nm
50 nm
50 nm
50 nm
B
# of fibers
Radius of curvature of fibers (nm)
C
# of fibers
Slope
D
100 nm
E
Slope values
100 nm
100 nm
100 nm
F
69°
20 nm
G
37°
64°
20 nm
H
31°
48°
20 nm
I
42°
20 nm
0.76 um deep
0.55 um deep

Figure S5 | The fibers travel straight and horizontally in the cell wall. Related to Figure 3.
(**A**) Examples of fibers with average radii of curvature spanning a range of 250 to 200nm. The fibers are highlighted in orange. (**B**) Global distribution of the average radius of curvature of all fibers across all scales, with an average at 225 ± 90 nm. (**C**) Global distribution of the average slopes of the fibers across all scales, with an average at 0.02 ± 0.4. (**D**) Segmented tomographic volume with color coding reflecting the slope of the fibers. Cyan fibers hold a slope value around 0. (**E**) Transversal views of small sub-volumes (boxed with the corresponding color in (D)) showing how the fibers are relative to the horizontal after lamella angle correction. (**F, G**) Tomographic slices showing events of highly curved fibers at 0.76 um below the plasma membrane. (**H, I**) Tomographic slices showing events of highly curved fibers at 0.55 um below the plasma membrane. The dashed lines are slightly shifted from the fiber densities in order for the latter to be seen.

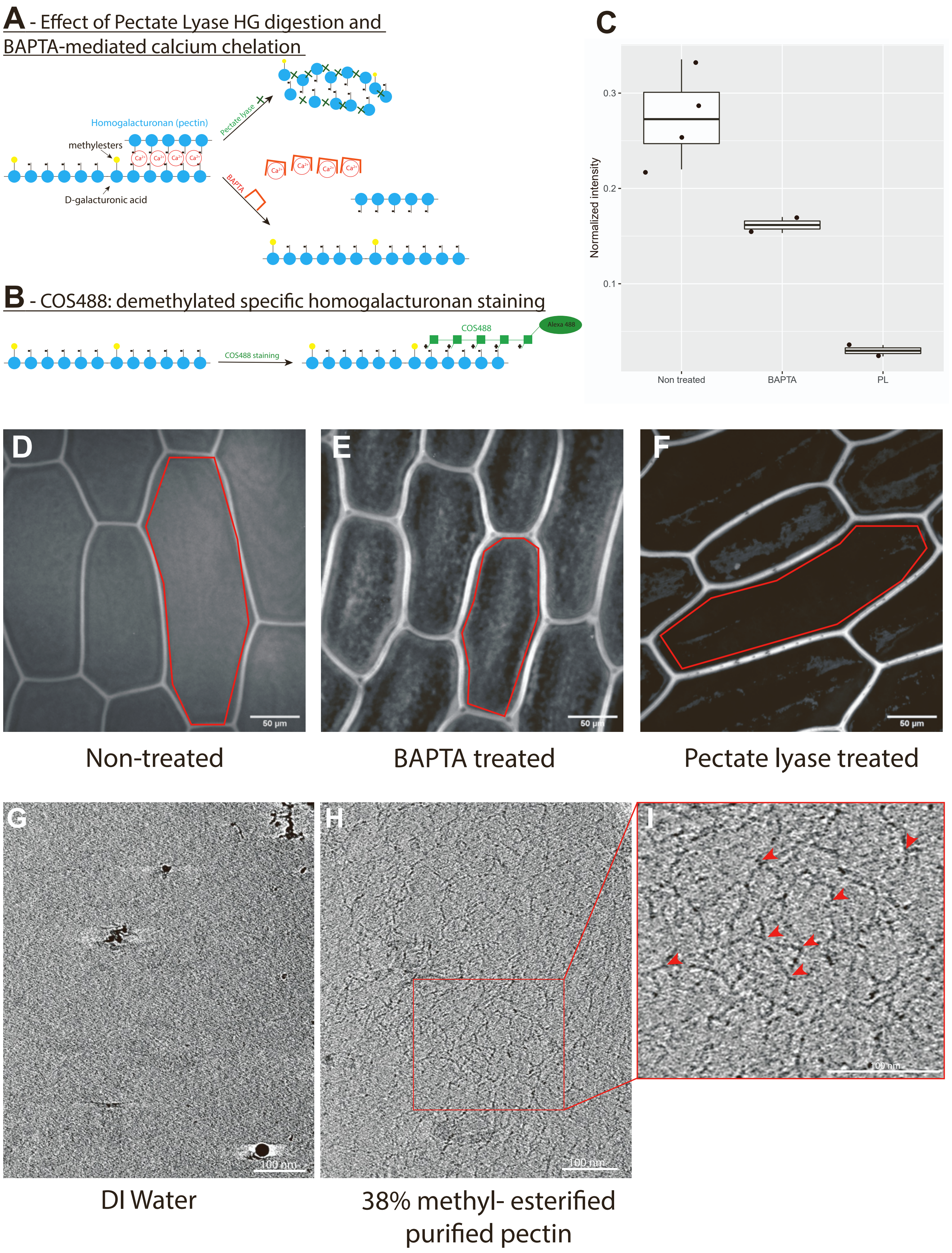

A - Effect of Pectate Lyase HG digestion and BAPTA-mediated calcium chelation
Homogalacturonan (pectin)
methylesters
D-galacturonic acid
Pectate lyase
BAPTA
B - COS488: demethylated specific homogalacturonan staining
COS488 staining
COS488
Alexa 488
C
Normalized intensity
0.3
0.2
0.1
Non treated
BAPTA
PL
D
E
F
50 µm
Non-treated
BAPTA treated
Pectate lyase treated
G
H
I
100 nm
DI Water
38% methyl- esterified purified pectin

Figure S6 | COS staining and effect of PL and BAPTA on HG pectins. Related to Figure 6 and STAR methods.

(**A**) Homogalacturonans demethylated groups can be cross-linked by the calcium present in the cell wall. Pectate lyase will sever the connections between the D-galacturonic acid units (top). BAPTA chelates the calcium, preventing cross-linking of pectins (bottom). (**B**) COS-488 is used to stain demethylated HGs by specifically binding to the demethylated groups. (**C**) Fluorescence intensity quantification of the effect of the PL and BAPTA treatments on the COS-488 stained peels. (**D**) Non-treated COS-488 stained peels showing homogeneous staining of the periclinal cell wall. (**E**) BAPTA-treated COS-488 stained peels showing a different, heterogeneous staining in the periclinal cell wall. (**F**) PL-treated COS-488 stained peels showing a significant decrease in the intensity of the signal in the periclinal cell wall. (**G**) Central tomographic slice from a tomogram of the distilled water used to dissolve pectins (negative control). (**H**) Central tomographic slice from a tomogram of a 0.25% purified citrus pectin with 38% of methyl-esterification, showing a reticulated network. (**I**) Magnified view from (H) showing details of the pectin meshing in the form of short, branched segments (red arrows).

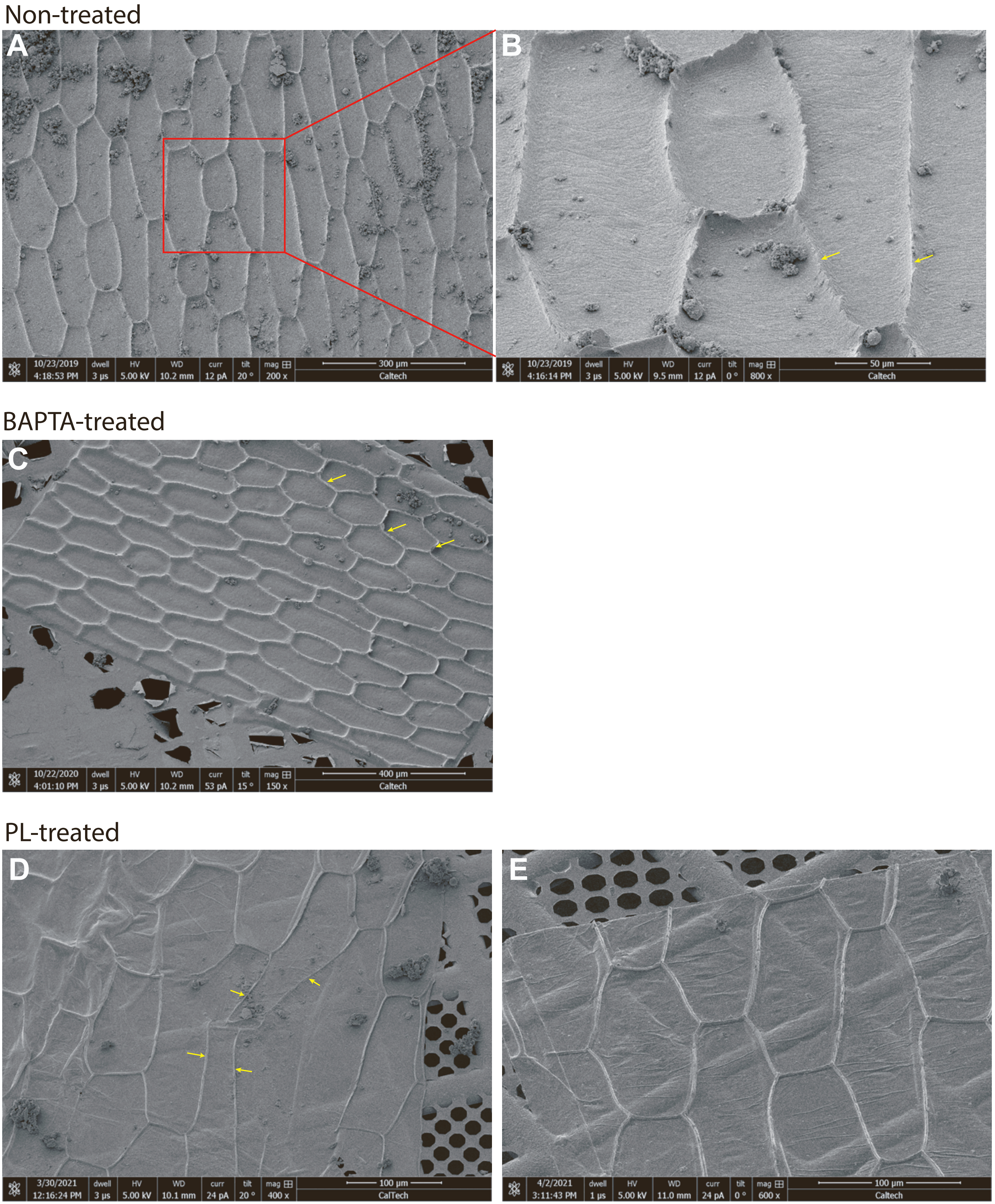
Non-treated
A
B
10/23/2019 4:18:53 PM dwell 3 μs HV 5.00 kV WD 10.2 mm curr 12 pA tilt 20 ° mag 200 x
300 μm
Caltech
10/23/2019 4:16:14 PM dwell 3 μs HV 5.00 kV WD 9.5 mm curr 12 pA tilt 0 ° mag 800 x
50 μm
Caltech
BAPTA-treated
C
10/22/2020 4:01:10 PM dwell 3 μs HV 5.00 kV WD 10.2 mm curr 53 pA tilt 15 ° mag 150 x
400 μm
Caltech
PL-treated
D
E
3/30/2021 12:16:24 PM dwell 3 μs HV 5.00 kV WD 10.1 mm curr 24 pA tilt 20 ° mag 400 x
100 μm
CalTech
4/2/2021 3:11:43 PM dwell 1 μs HV 5.00 kV WD 11.0 mm curr 24 pA tilt 0 ° mag 600 x
100 μm
Caltech

Figure S7 | Effect of PL and BAPTA treatment on the morphology of the onion cell wall peels. Related to Figure 6.

(**A**) SEM overview of a non-treated cell wall peel. (**B**) Magnified view of red rectangle in (A) showing the details of the periclinal cell wall and the anticlinal cell wall remains (yellow arrows). (**C**) SEM overview of a BAPTA-treated cell wall peel with a similar morphology to the non-treated cell wall peel. Yellow arrows point out to the anticlinal cell wall remains. (**D**) SEM overview of a PL-treated cell wall peel showing a thinned-out periclinal cell wall and detached anticlinal remains (yellow arrows). (**E**) Additional example of a PL-treated cell wall peel showing how thin they are compared to non-treated and BAPTA-treated peels.

Condition	Individual onion # (freezing time)	Scale	Tomograms
Non-treated	-	-	32
	6 (January 2021)	2	8
	2 (January 2020)	5	10
	1 (December 2018)	6	6
	3 (February 2020)	8	8
Pectate lyase-treated	-	-	6
	5 (November 2020)	5	5
	5 (November 2020)	6	1
BAPTA-treated	-	-	7
	4 (August 2020)	7	7
Total	6 onions over the course of 2 years	5	45

Table S1 | Summary of the tilt-series collected. Related to Figure 3 and 6.
Break down of the tilt-series collected by condition (1st column), provenance (2nd column, onions were numbered from 1 to 6) and scale where the lamellae were milled (3rd columns).
